# Primary and secondary tumors of the peritoneum: key imaging features and differential diagnosis with surgical and pathological correlation

**DOI:** 10.1186/s13244-023-01417-6

**Published:** 2023-07-03

**Authors:** Javier Miguez González, Francesc Calaf Forn, Laura Pelegrí Martínez, Pilar Lozano Arranz, Rafael Oliveira Caiafa, Jordi Català Forteza, Lina Maria Palacio Arteaga, Ferrán Losa Gaspà, Isabel Ramos Bernadó, Pedro Barrios Sánchez, Juan Ramón Ayuso Colella

**Affiliations:** 1 Department of Radiology, Complex Hospitalari Universitari Moisès Broggi, Consorci Sanitari Integral, Sant Joan Despí, Barcelona Spain; 2 Department of Pathology, Complex Hospitalari Universitari Moisès Broggi, Consorci Sanitari Integral, Sant Joan Despí, Barcelona Spain; 3 Department of Medical Oncology, Institut Català d’Oncologia Hospitalet, Complex Hospitalari Universitari Moisès Broggi, Consorci Sanitari Integral, Barcelona, Spain; 4 Peritoneal Surface Malignancies Unit, Department of Surgery, Complex Hospitalari Universitari Moisès Broggi, Consorci Sanitari Integral, Sant Joan Despí, Barcelona Spain; 5 Former Director of the Peritoneal Carcinomatosis Program of Catalonia, Former Head of the Peritoneal Surface Malignancies Unit, Department of Surgery, Complex Hospitalari Universitari Moisès Broggi, Consorci Sanitari Integral, Sant Joan Despí, Barcelona, Spain; 6 Department of Radiology, Hospital Clinic, Barcelona, Spain

**Keywords:** Imaging of peritoneal tumors, Peritoneal carcinomatosis, Pseudomyxoma peritonei, Peritoneal sarcomatosis, Peritoneal mesothelioma

## Abstract

Peritoneal malignancies represent a diagnostic challenge for abdominal radiologists, oncologists, surgeons and pathologists in multidisciplinary teams, who must address their differential diagnosis, staging and treatment. In this article, we explain the pathophysiology of these processes and lay out the role of different imaging techniques in their evaluation. Then, we review the clinical and epidemiological aspects, the main radiological features and the therapeutic approaches for each primary and secondary peritoneal neoplasm, with surgical and pathological correlation. We further describe other rare peritoneal tumors of uncertain origin and a variety of entities that may mimic peritoneal malignancy. Finally, we summarize the key imaging findings of each peritoneal neoplasm to facilitate an accurate differential diagnosis that may impact patient management.

**Clinical relevance statement**

Imaging plays an essential role in the evaluation of peritoneal malignancies, assessing their extension, detecting unfavorable sites of involvement and facilitating an accurate differential diagnosis, helping to choose the best therapeutic approach.

**Graphical abstract:**

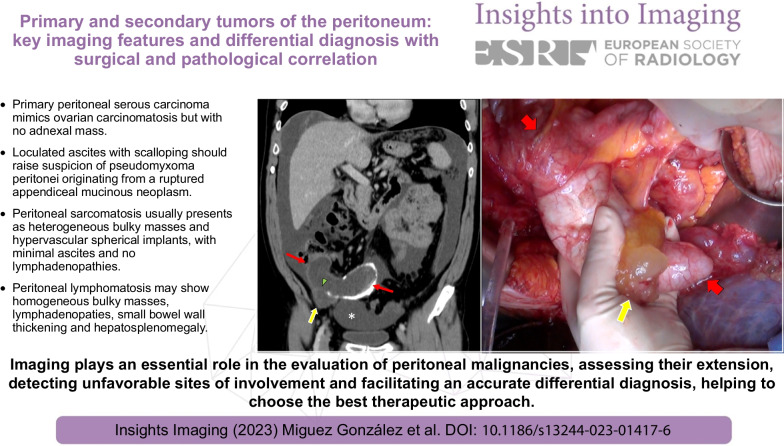

## Key points

- The most frequent peritoneal malignancy is peritoneal carcinomatosis of ovarian or gastrointestinal origin.
- Primary peritoneal serous carcinoma mimics ovarian carcinomatosis but with no adnexal mass.
- Loculated ascites with scalloping should raise suspicion of pseudomyxoma peritonei originating from a ruptured appendiceal mucinous neoplasm.
- Peritoneal sarcomatosis usually presents as heterogeneous bulky masses and hypervascular spherical implants, with minimal ascites and no lymphadenopathies.
- Peritoneal lymphomatosis may show homogeneous bulky masses, lymphadenopathies, small bowel wall thickening and hepatosplenomegaly.

## Introduction

Peritoneal malignancies represent a heterogeneous group of neoplasms with different characteristics in terms of incidence, diagnosis, treatment options and prognosis [1].

Their clinical presentation is insidious and often delays the diagnosis, as many patients are asymptomatic in its early stages or complain of  non-specific symptoms such as abdominal distension and discomfort due to ascites. As the disease progresses, symptoms become more evident, causing more intense abdominal pain with nausea, vomiting and eventually bowel obstruction [1–6].

Although traditionally considered fatal diseases, the development of new therapeutic strategies in recent years, such as the combination of cytoreductive primary surgery (CRS) with hyperthermic intraperitoneal chemotherapy (HIPEC), has changed this scenario, leading to an overall improvement in patient survival [1, 5].

The main purpose of this work is to carry out a comprehensive review of the broad spectrum of peritoneal tumors, emphasizing those key imaging features useful for differential diagnosis and correlating them with the surgical and pathological findings.

## Pathophysiology of peritoneal malignancies

Primary peritoneal tumors are rare neoplasms that arise from the malignant transformation of cells located in the mesothelial or submesothelial layers of the peritoneum [1, 2].

**Fig. 1:**
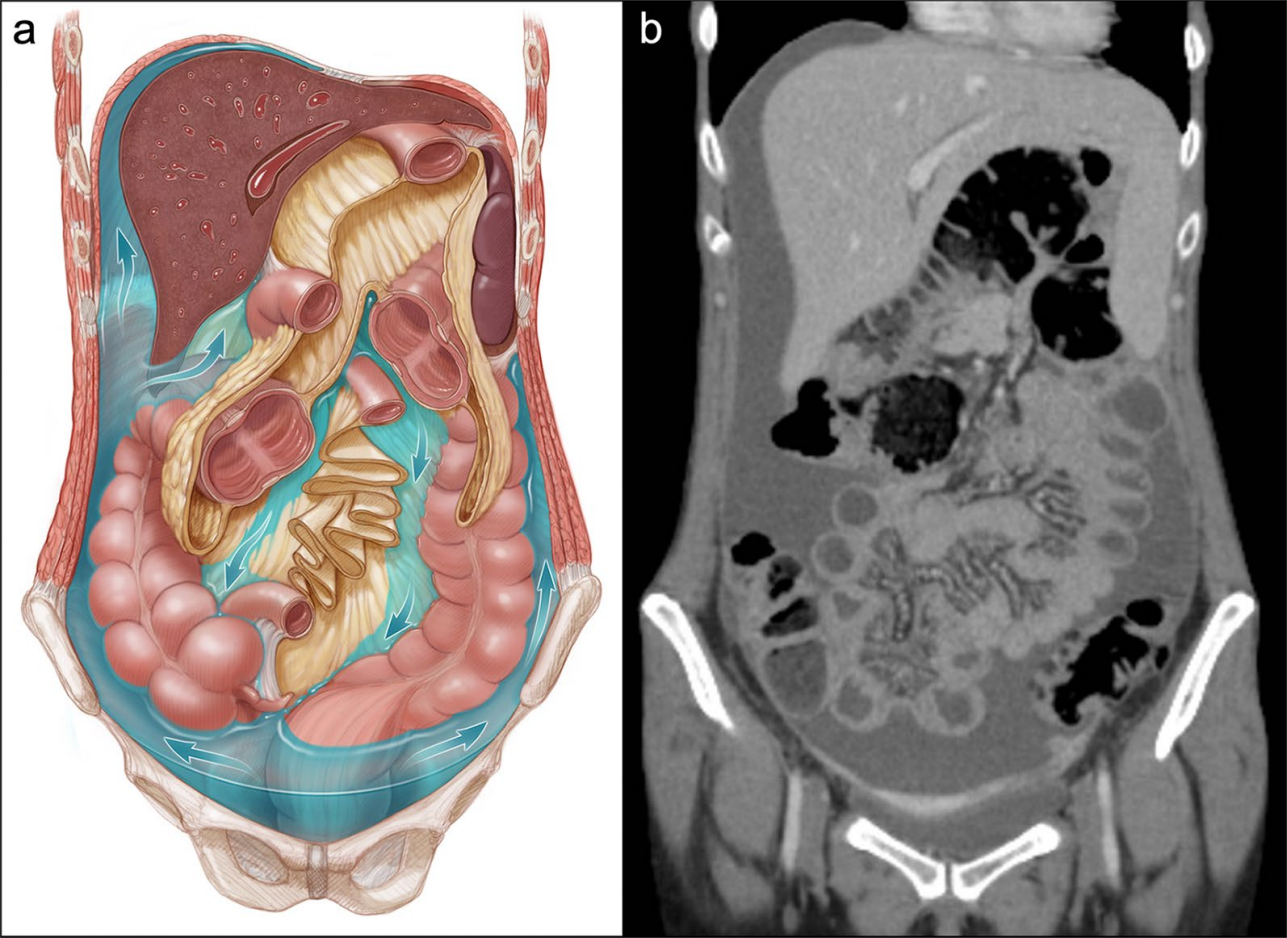
Flow dynamics of peritoneal fluid. **a** Illustration of the abdominal cavity in coronal plane, depicting the peritoneal ligaments and mesenteries and the pathways of ascitic fluid (blue arrows). Initially, the fluid is collected in the pelvic recesses due to gravity. Then, and due to a pressure gradient, it flows upward mainly along the right paracolic gutter toward the Morison pouch and the right subphrenic space. The fluid also flows upward along the left paracolic gutter but to a lesser degree, as it is a shallow and discontinuous space limited superiorly by the phrenicocolic ligament. **b** Coronal CT image of a patient with peritoneal dissemination secondary to an advanced gastric cancer, showing the predominant distribution of ascitic fluid in the pelvis, right paracolic gutter and right subphrenic space

Secondary tumors of the peritoneum show a significantly higher prevalence and represent an advanced evolutive stage of metastatic neoplasms, known under the generic term of peritoneal carcinomatosis. This condition is usually secondary to intra-abdominal neoplasms, mainly ovarian and gastrointestinal cancers [1, 3–6], although occasionally extra-abdominal neoplasms like breast cancer, lung cancer or malignant melanoma may also metastasize to the peritoneum [1, 7]. Four pathways have been described that, in isolation or synchronously, allow for this peritoneal dissemination [1, 3–5, 8]:

1. *Intraperitoneal seeding* This route is conditioned by the peritoneal ligaments and mesenteries, which dictate the flow dynamics of ascitic fluid [8]. In patients with ascites related to an underlying malignant process, the fluid tends to collect in well-defined areas like the pelvic recesses, the superior aspect of the sigmoid mesocolon, the inferior part of the small bowel mesentery, the ileocecal junction, the right paracolic gutter, the Morison pouch and the right subphrenic space (Fig. 1). These areas of stasis facilitate the transcoelomic dissemination of free-floating neoplastic cells, which detach from the surface of the primary tumor and adhere to the peritoneal sheets [1, 3–5, 8].
2. *Direct invasion* In this route intra-abdominal neoplasms, like gastric carcinomas and small bowel neuroendocrine tumors, cross the serous membrane to invade directly the adjacent ligaments and mesenteries [3–5].
3. *Lymphatic extension* Tumoral cells circulating in the lymphatic vessels may reach the peritoneum through lymphatic stomata located on the surface of the diaphragm. This pathway plays a major role in non-Hodgkin lymphomas and is also frequently seen in ovarian and gastrointestinal cancers [3–5].
4. *Embolic hematogenous spread* Aggressive neoplasms that invade vascular walls may reach the peritoneal surfaces via the bloodstream. This route is typical of peritoneal metastases from extra-abdominal primary tumors [3–5].

## Role of imaging in the evaluation of peritoneal tumors

Different imaging techniques are used in the study of peritoneal malignancies:

- Multidetector CT is the primary imaging modality to assess the presence and extension of peritoneal disease and to rule out extraperitoneal metastases, due to its widespread availability and high speed of acquisition [1, 4, 6, 9]. However, several studies have shown that this technique often underestimates the volume of peritoneal disease with respect to surgical evaluation [10–13].
- PET/CT is a highly accurate technique for ruling out nodal and extraperitoneal disease and detecting recurrences that may go unnoticed on CT, with [18F] FDG being the most common radiotracer. Nevertheless, it may show false-negative results in small-size peritoneal implants, mucinous tumors or signet ring gastric cancers, and false-positive results in non-malignant inflammatory lesions [1, 4].
- Peritoneal MRI has emerged as a promising alternative imaging tool for staging and surveillance. It provides excellent soft tissue contrast and allows multiphasic contrast-enhanced imaging and diffusion-weighted imaging, thus facilitating the detection of disease in challenging sites like the mesentery and the small bowel serosa [1, 4, 10, 13–15]. However, the limited availability of this technique associated with longer examination time, motion artifacts and lack of experience in interpretation among radiologists and surgeons limits its broader implementation [1, 4, 13].
- Ultrasound plays a minor role in the evaluation of peritoneal tumors, but it is useful to identify malignant ascites and is an optimal modality for image-guided biopsy when histological diagnosis is required [1, 4, 6, 16].

**Fig. 2:**
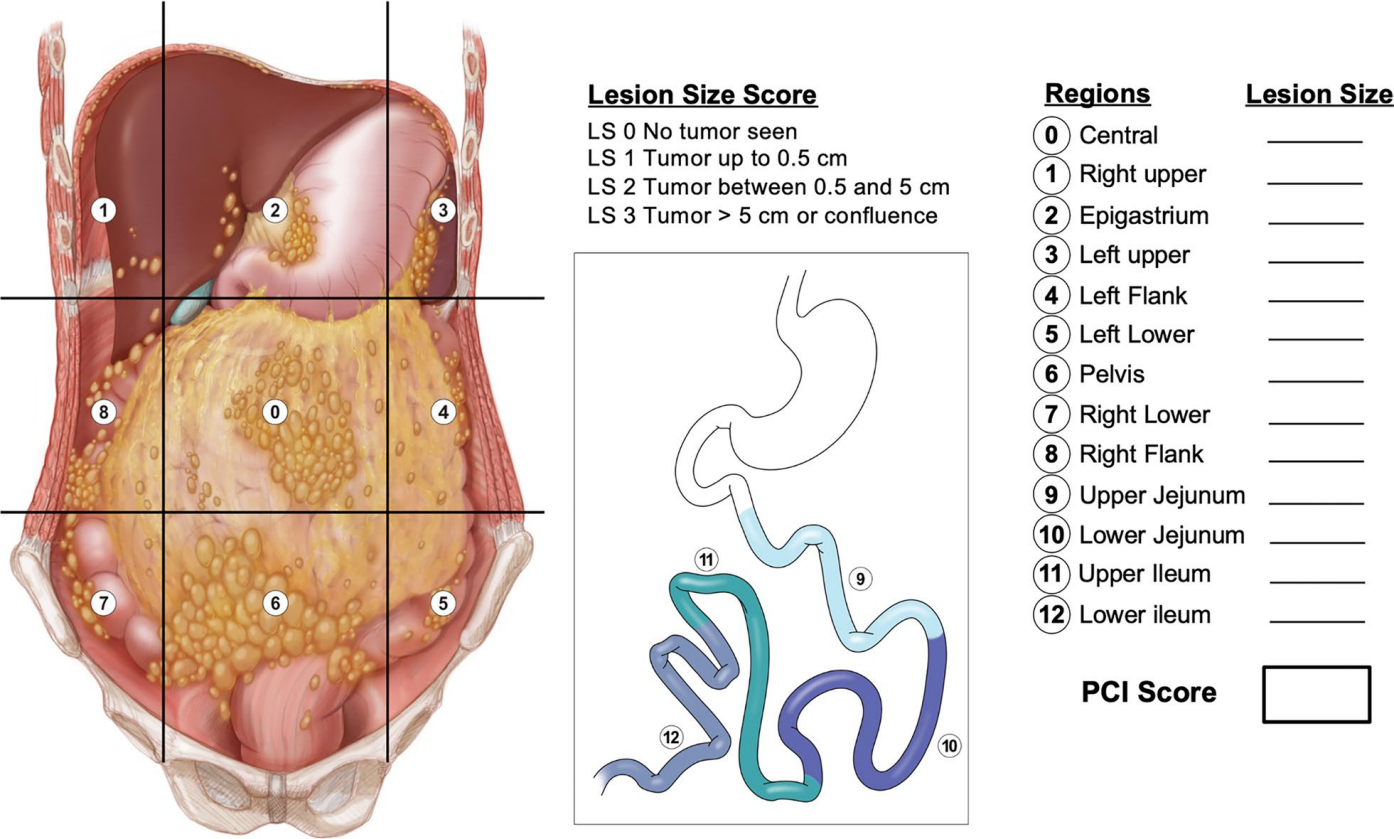
Illustrations of the abdominal cavity and the small bowel in coronal plane, showing the distribution of peritoneal metastases according to the Peritoneal Cancer Index (PCI) originally described by Jacquet and Sugarbaker [17]

In order to facilitate the intraoperative assessment of the volume and extent of peritoneal disease, Jacquet and Sugarbaker described an original system called Peritoneal Cancer Index (PCI) [17], which has subsequently been adapted for radiological purposes. It divides the peritoneum into thirteen sites for assessment: the abdomen and pelvis are divided into nine sites and the small bowel loops (jejunum and ileum) are divided into other four parts. The size of tumor deposits is assessed individually in each site, with a score that ranges from 0 to 3. The total PCI score is calculated by adding together the scores for each region, with a minimum score of 0 and a maximum score of 39 (Fig. 2).

**Fig. 3:**
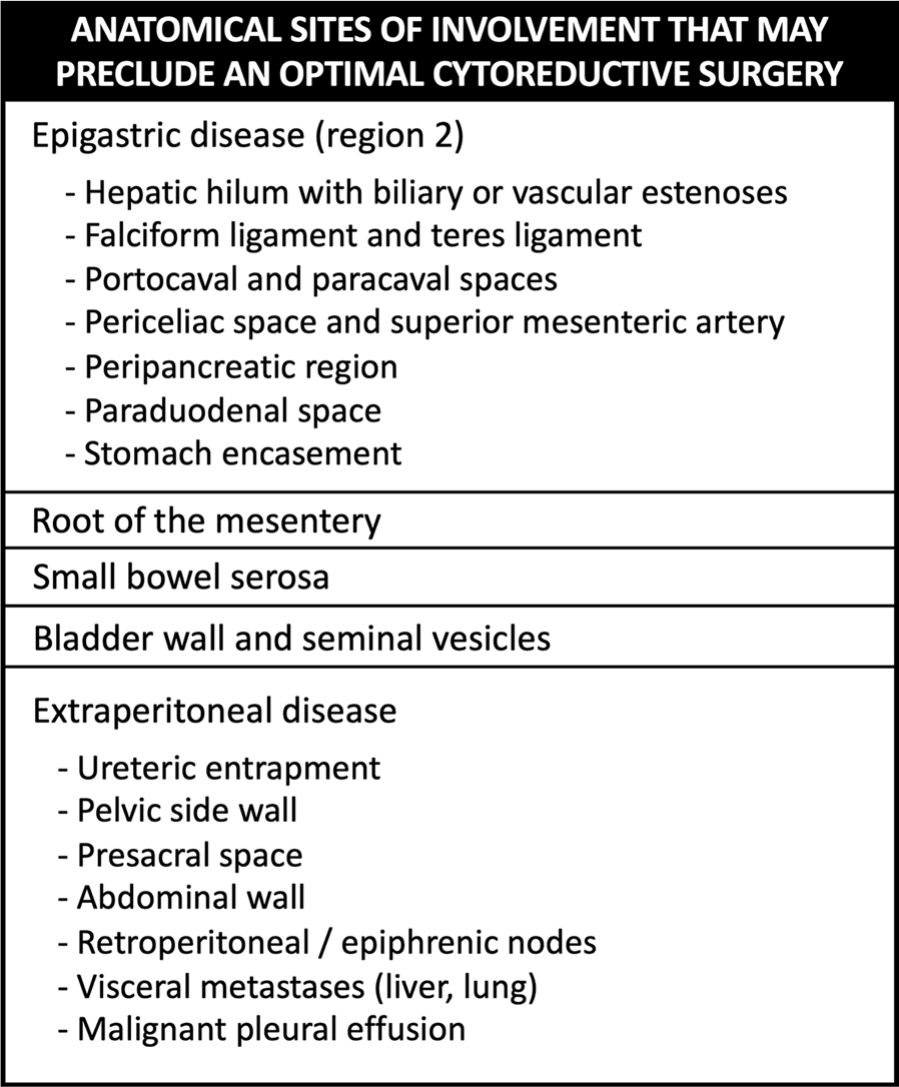
Anatomical sites of involvement in peritoneal tumors that may prevent a complete cytoreductive surgery (CRS)

Since imaging techniques tend to underestimate the extent of peritoneal disease, the main role of the radiologist is not to calculate the exact PCI, but rather to provide an overall assessment of the tumor burden and to identify those anatomical sites of involvement that may preclude a complete CRS (Fig. 3), thus guiding the best therapeutic approach [15, 18, 19].

A standardized radiological report is still a pending task. Interesting efforts in this direction have been made by Low et al. [15] and by Chandramohan et al. [18, 19] with its acronym “PAUSE” (Primary tumor and PCI; Ascites and abdominal wall involvement; Unfavorable sites of involvement; Small bowel and mesenteric disease; Extraperitoneal metastases), which aims to make a structured description of the key imaging findings that impact surgical decision-making. Villeneuve et al. [20] have also developed a useful Internet application called PROMISE, which facilitates the calculation of the PCI score.

## Classification of peritoneal tumors

**Fig. 4:**
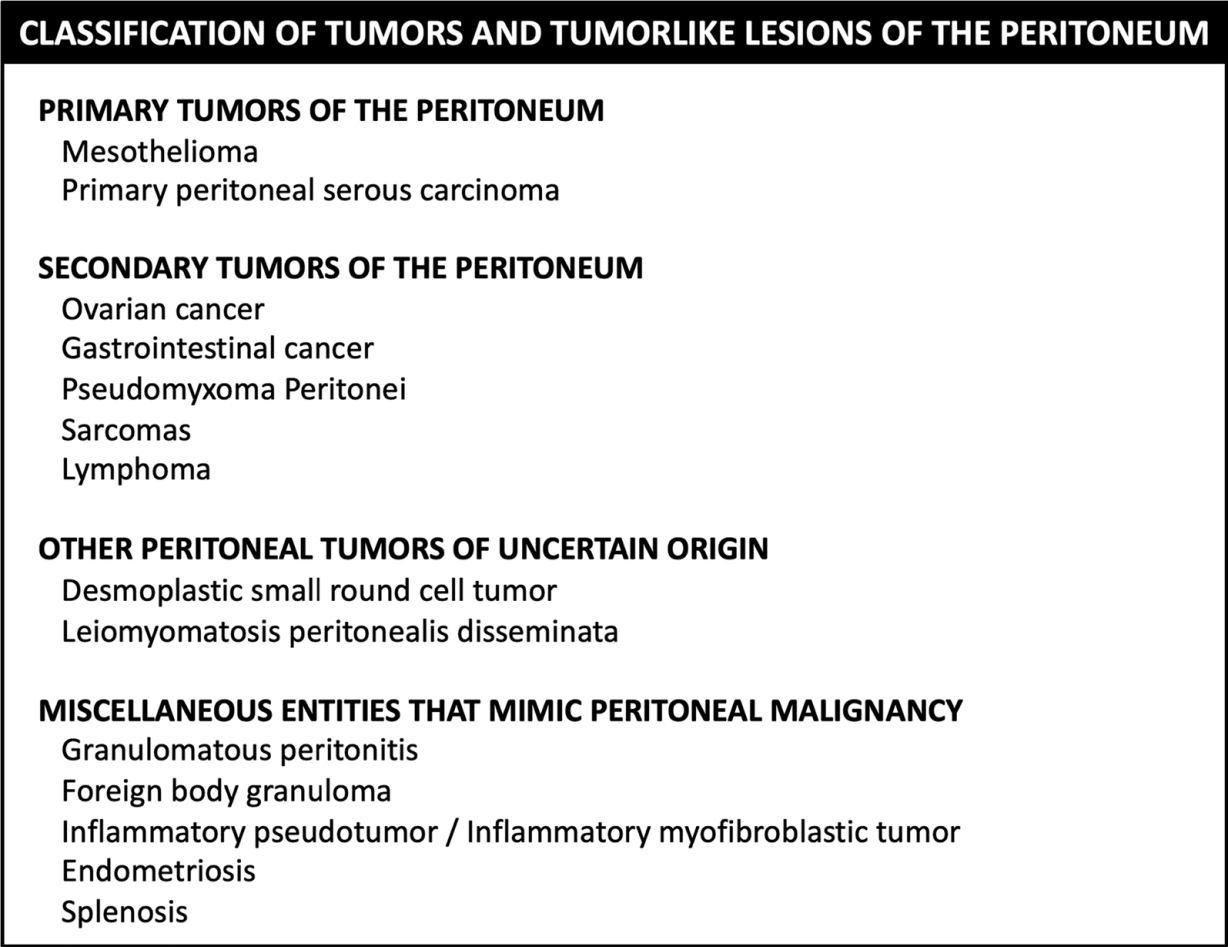
Classification of tumors and tumorlike lesions of the peritoneum

Peritoneal neoplasms can be classified into primary and secondary tumors, although there are a few rare peritoneal tumors of uncertain origin. Moreover, some benign entities may mimic peritoneal malignancy (Fig. 4).

## Primary tumors of the peritoneum

### Mesothelioma

Mesothelioma is a rare neoplasm arising from the mesothelial cells that form the serosal membranes of the body cavities. The peritoneal cavity is the second most common site involved after the pleura, and it is affected in approximately 10% to 30% of cases, either solely or in combination with pleural involvement [19, 21, 22].

There are three main types of peritoneal mesothelioma with different imaging features: malignant peritoneal mesothelioma (MPM), well-differentiated papillary mesothelioma (WDPM) and benign multicystic mesothelioma (BMM) [21].

#### Malignant peritoneal mesothelioma

MPM is the most frequent type of mesothelioma and affects mainly men in the fifth and sixth decades of life. Association with asbestos exposure can be documented in approximately half of the cases. It can be subclassified into three different histologic types: epithelioid, sarcomatoid and biphasic [19, 21, 22].

**Fig. 5:**
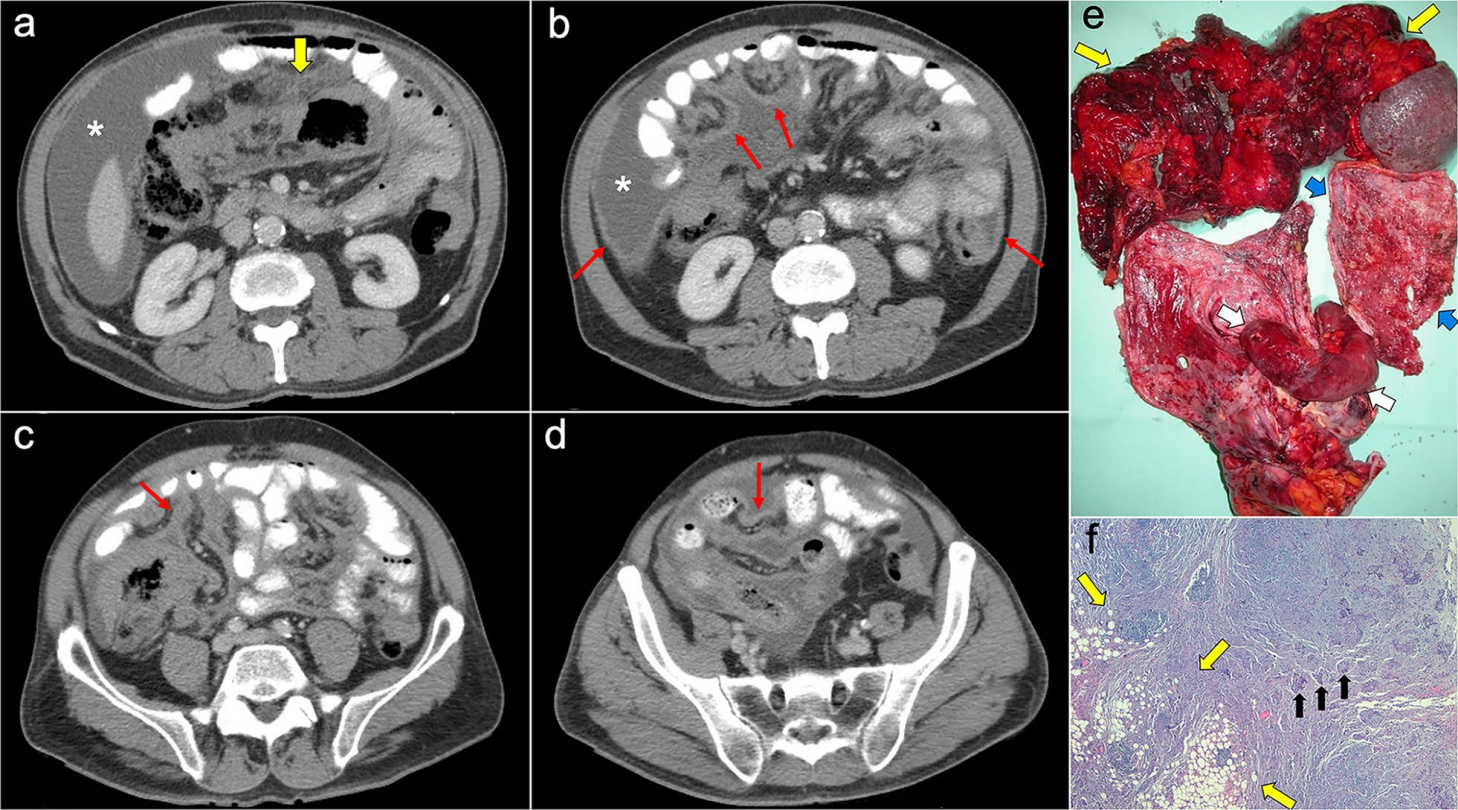
Malignant epithelioid peritoneal mesothelioma in a 60-year-old man. **a**–**d** Axial CT images with positive oral contrast and intravenous contrast in the portal phase show “wet type” malignant epithelioid mesothelioma with ascites (white asterisks), stranding of the mesenteric fat (yellow arrow) and diffuse thickening of the peritoneal folds more prominent in the mesenteric root (red arrows). **e** Surgical specimen shows multiple implants with a diffuse distribution involving the parietal peritoneum (blue arrows), the greater omentum (yellow arrows), the small bowel mesentery, rectum and sigma (white arrows). **f** Hematoxylin–eosin (H&E) stain photomicrograph shows a malignant epithelioid peritoneal mesothelioma with papillary areas (black arrows), that infiltrates the adjacent soft tissues (yellow arrows)

**Fig. 6:**
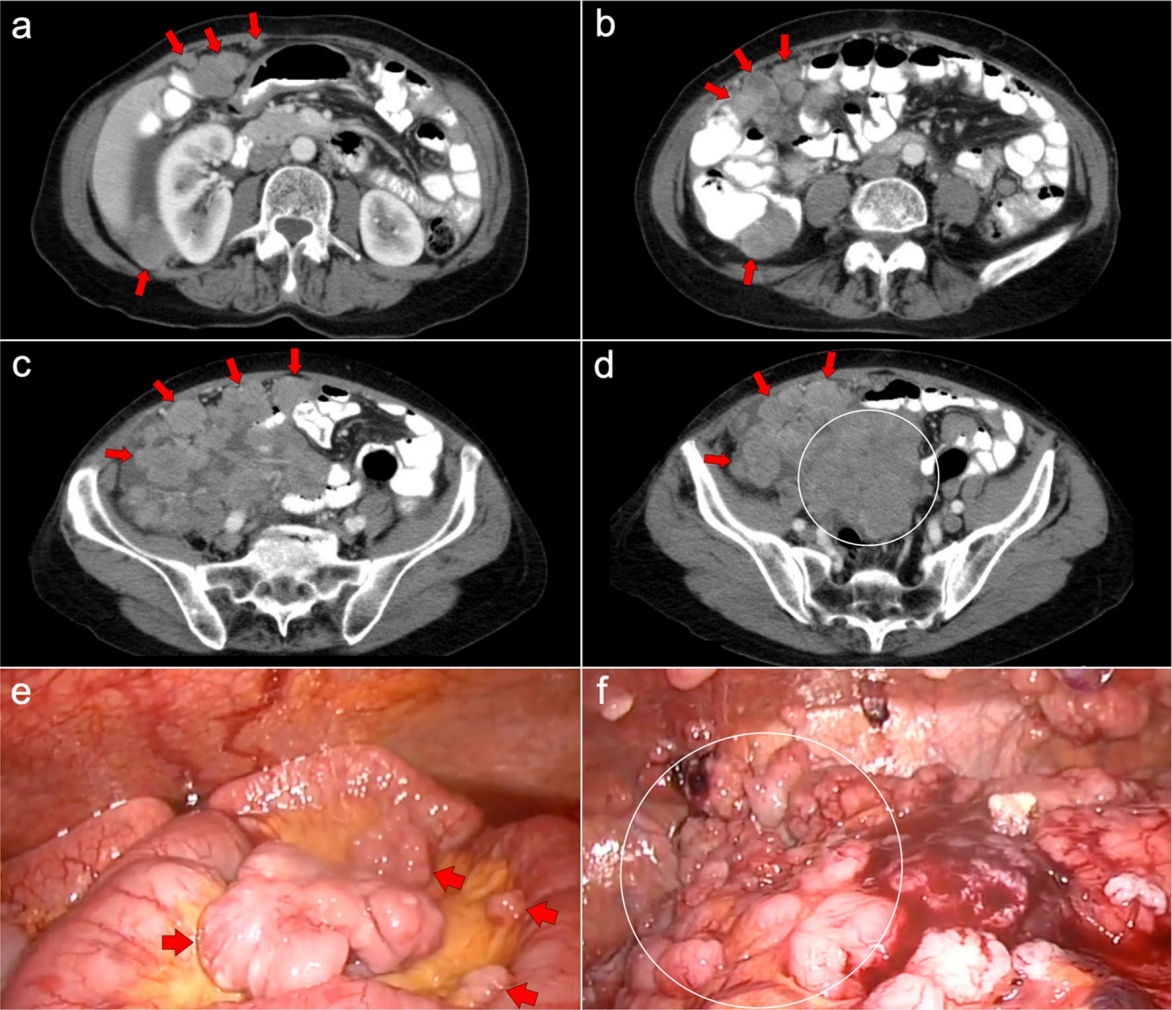
Malignant sarcomatoid peritoneal mesothelioma in a 69-year-old woman. **a–d** Axial CT images with positive oral contrast and intravenous contrast in the portal phase show a “dry type” malignant sarcomatoid mesothelioma, presenting with minimal ascites and multiple solid intraperitoneal lesions (red arrows) that converge into a bulky mass at the mesenteric root (white circle). **e, f** Laparoscopic images show several solid implants in the small bowel mesentery (**e**, red arrows) and multiple implants in the pelvis that converge in the inferior portion of the mesenteric root (**f**, white circle)

Imaging features depend on the histologic subtype. Epithelioid mesothelioma is the most frequent one and has a “wet appearance” that mimics peritoneal carcinomatosis, showing ascites, diffuse plaque-like peritoneal thickening, infiltration of the mesentery with fixation of bowel loops and omental cake (Fig. 5). Sarcomatoid mesothelioma is less common and more aggressive, showing a “dry appearance” with intraperitoneal solid masses and minimum ascites that mimics peritoneal sarcomatosis (Fig. 6). The biphasic subtype is a combination of the two previous subtypes [19, 21].

Positive staining for immunohistochemical markers like vimentin, calretinin, cytokeratin 5/6, podoplanin and WT-1 is characteristic of mesotheliomas and is essential to distinguish them from secondary tumors of the peritoneum [19, 22, 23]. Prognosis is poor, although a combination of CRS with HIPEC has achieved a clear improvement in survival in comparison with systemic chemotherapy, with multi-institutional studies reporting a 5-year survival rate between 41 and 47% [24, 25].

#### Well-differentiated papillary mesothelioma

This is a very rare type of mesothelioma that often arises from the peritoneal surfaces of the pelvis, typically in reproductive-age women with no predisposing factors and no evidence of asbestos exposure. It is frequently asymptomatic and often discovered incidentally during pelvic surgery [2, 19, 21, 26].

**Fig. 7:**
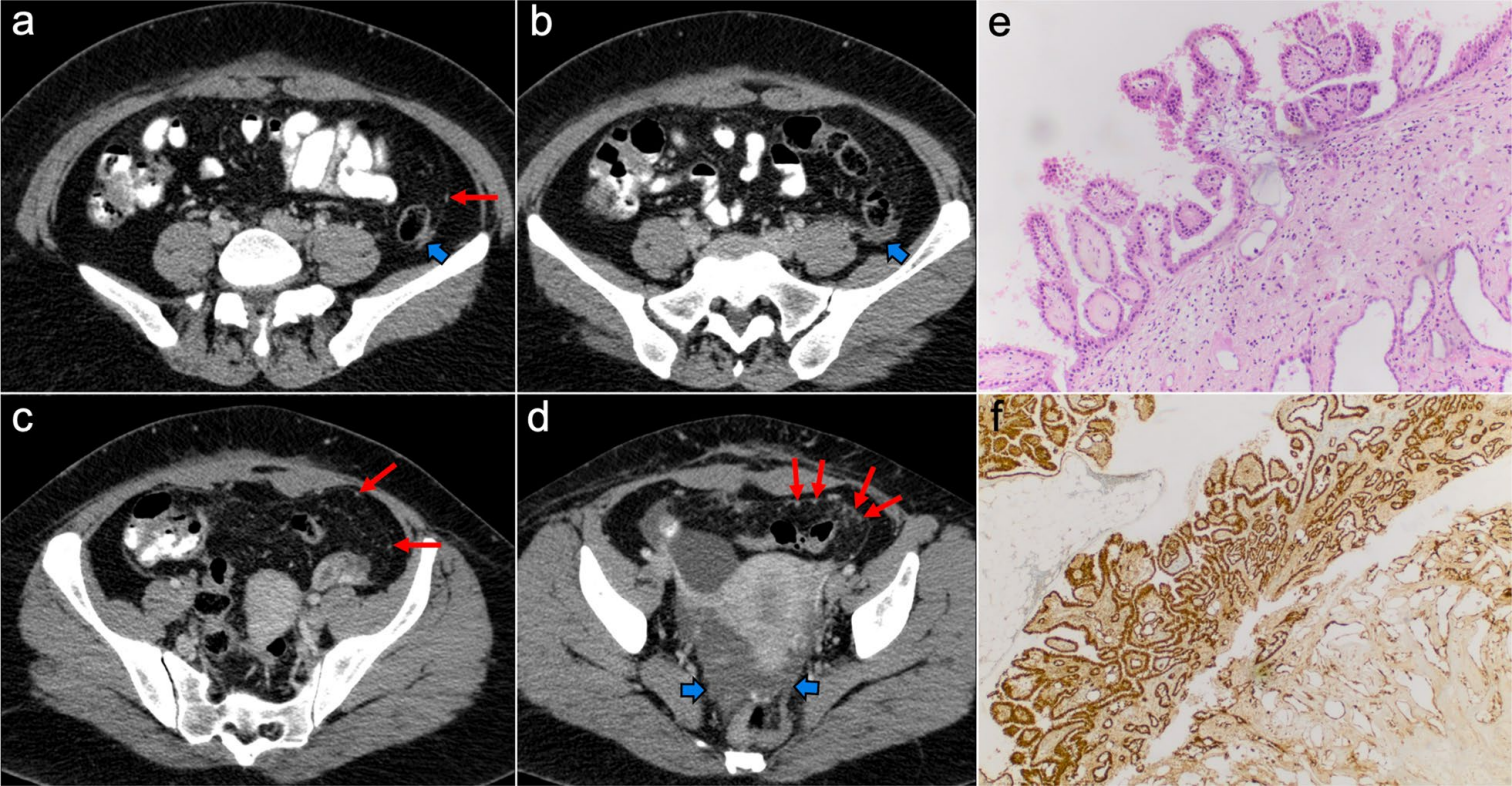
Well-differentiated papillary mesothelioma in a 39-year-old woman. **a–d** Axial CT images in the portal phase show tiny peritoneal nodules on the left paracolic gutter and anterior pelvic fascia (red arrows), associated with a small amount of ascitic fluid (blue arrows). H&E stain (**e**) and calretinin immunohistochemical staining (**f**) photomicrographs of the surgical biopsy show branching papillae covered by a single layer of calretinin-positive mesothelial cells, with no cellular atypia

Imaging of WDPM has rarely been described in the radiological literature because of its lack of specific features. It may mimic peritoneal carcinomatosis with ascites, peritoneal thickening, peritoneal-based nodules and omental infiltration. Nevertheless, the disease may be challenging to detect on CT, PET/CT or MRI, given that more than half of the reported cases are composed of tiny millimeter-sized nodules (Fig. 7). In some cases, psammomatous calcifications may be present [2, 19, 21].

The final diagnosis is histological, characterized by uniform coarse or branching papillae covered by a single layer of mesothelial cells, with mild or absent cellular atypia [21, 26]. The prognosis is good, as it is a tumor of low malignant potential that is usually cured after complete surgical resection or follows an indolent course with long survival [2, 26].

#### Benign multicystic mesothelioma

BMM, also known as peritoneal inclusion cyst, is a rare type of mesothelioma that arises from the peritoneal surfaces of the pelvis. It is frequently seen in young to middle-aged women and has no association with asbestos exposure. The exact pathogenesis of this entity remains unclear. Some authors advocate for chronic peritoneal irritation, as there is often a history of previous surgery, pelvic inflammatory disease or endometriosis. Other authors believe in a neoplastic origin of the lesion, given the reported tendency to local recurrence after surgery [27, 28].

**Fig. 8:**
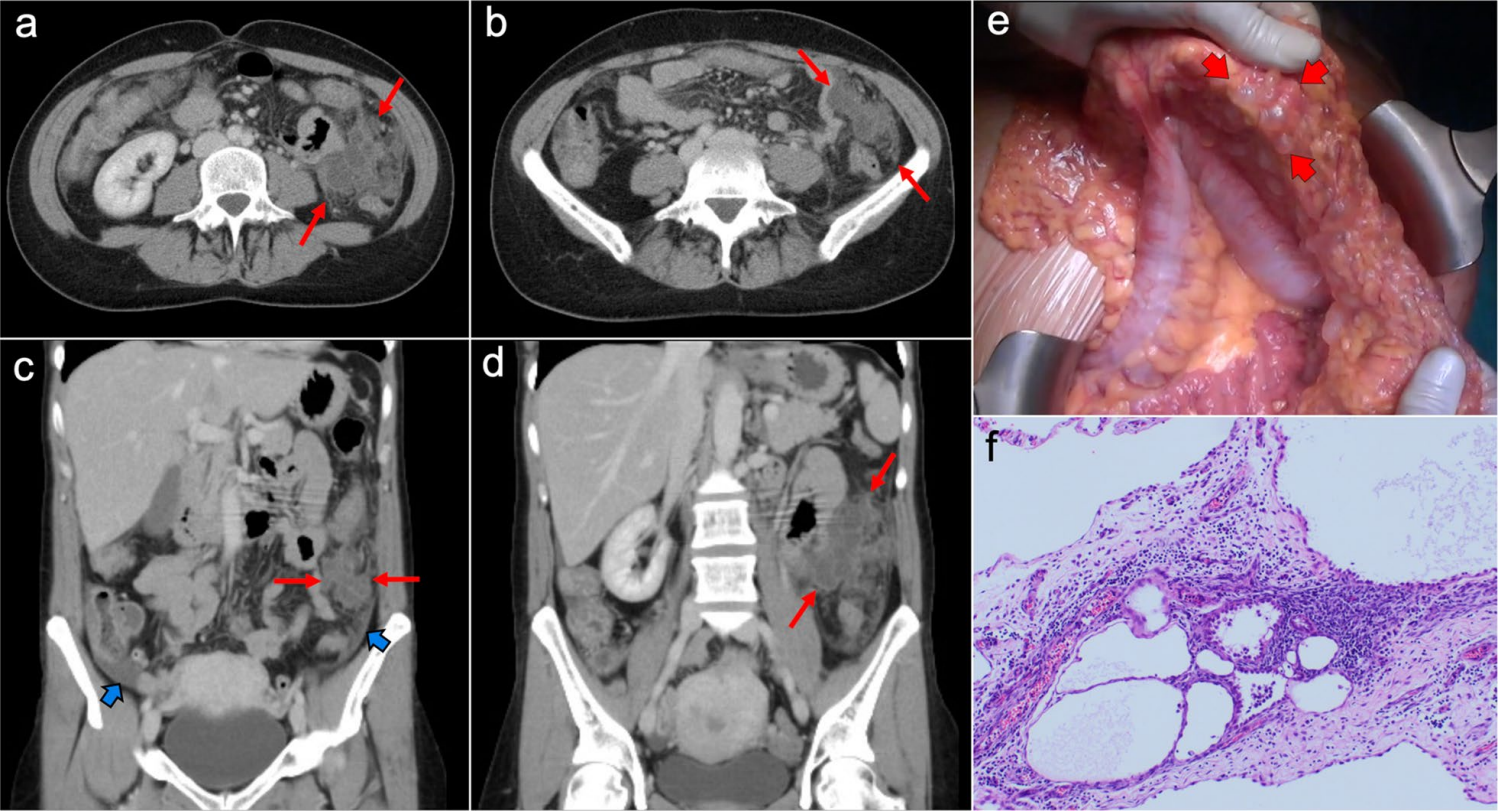
Benign multicystic peritoneal mesothelioma in a 41-year-old woman with history of ulcerative colitis and left flank pain. Axial (**a**, **b**) and coronal (**c**, **d**) CT images in the portal phase show cystic confluent lesions of grapelike appearance on the left paracolic gutter (red arrows), with stranding of adjacent omental fat and a small amount of ascitic fluid (blue arrows). **e** Intraoperative view demonstrates clusters of small thin-walled cysts in the greater omentum with a grapelike morphology. **f** H&E stain photomicrograph of the surgical biopsy shows multiple cystic spaces with hyaline fibrous septa, lined by sheets of epithelium with mesothelial cells

**Fig. 9:**
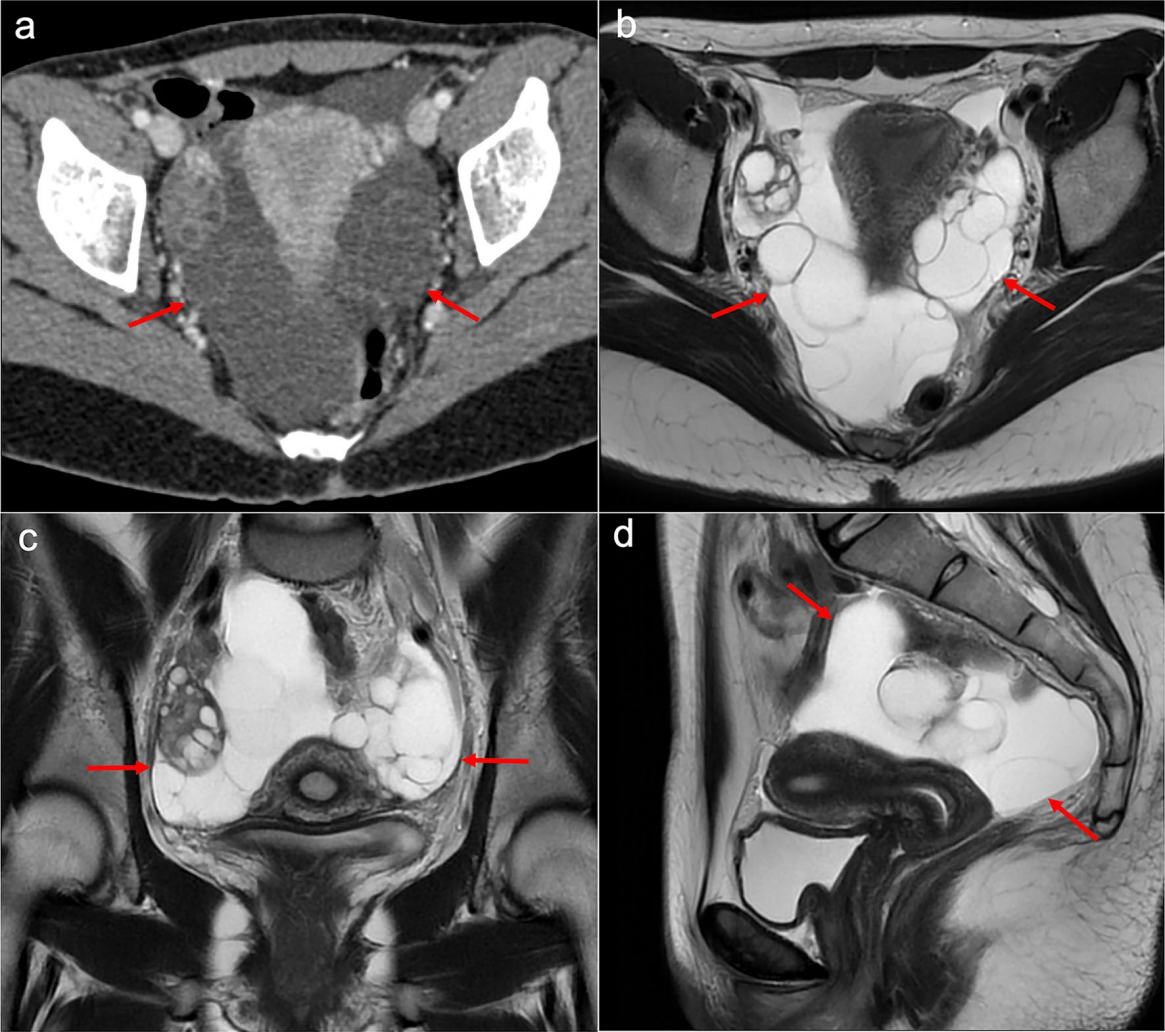
Benign multicystic peritoneal mesothelioma in a 21-year-old woman with abdominal pain. **a** Axial CT image in the portal phase shows a hypodense multilocular pelvic mass of cystic appearance with thin septa (red arrows). T2-weighted MR images of the pelvis in axial (**b**), coronal (**c**) and sagittal (**d**) planes confirm the presence of a hyperintense multilocular cystic pelvic mass surrounding the uterus and ovaries (red arrows), containing clusters of thin-walled cysts with a grapelike morphology. The suspected diagnosis of a benign multicystic peritoneal mesothelioma was confirmed after laparoscopic surgery

The CT and MRI appearance consists of multilocular cystic masses or multiple unilocular thin-walled cysts distributed along the pelvic peritoneum and the paracolic gutters in grapelike clusters (Figs. 8, 9). Mild ascites and minimal peritoneal thickening may be present. The differential diagnosis includes cystic lymphangiomas, mesenteric cysts, paraovarian cysts, endometriosis and pseudomyxoma [19, 27, 28].

At histological analysis, BMM shows multiple small cystic spaces with hyaline fibrous septa, lined by a single layer of calretinin-positive mesothelial cells, without atypical features or tissue invasion [27, 28].

Treatment remains controversial, as there are no evidence-based guidelines available. An optimal CRS with complete excision is the treatment of choice in most cases, although some authors have also suggested a combination of CRS with HIPEC or conservative management [27–29]. Despite frequent local recurrence, the prognosis of BMM is very good, with no metastatic potential and very few reports describing malignant transformation [27].

### Primary peritoneal serous carcinoma

Primary peritoneal serous carcinoma (PPSC), also known as primary peritoneal papillary serous carcinoma or primary peritoneal carcinoma, is an epithelial tumor almost exclusive of women in their fifth and sixth decades of life. It is a neoplasm that diffusely invades the peritoneal surface, but spares the ovaries or invades them only superficially [2, 30, 31]. It is thought to arise from extraovarian mesothelium that has Müllerian potential, making it a unique clinicopathological entity distinct from its ovarian counterpart [2, 32].

**Fig. 10:**
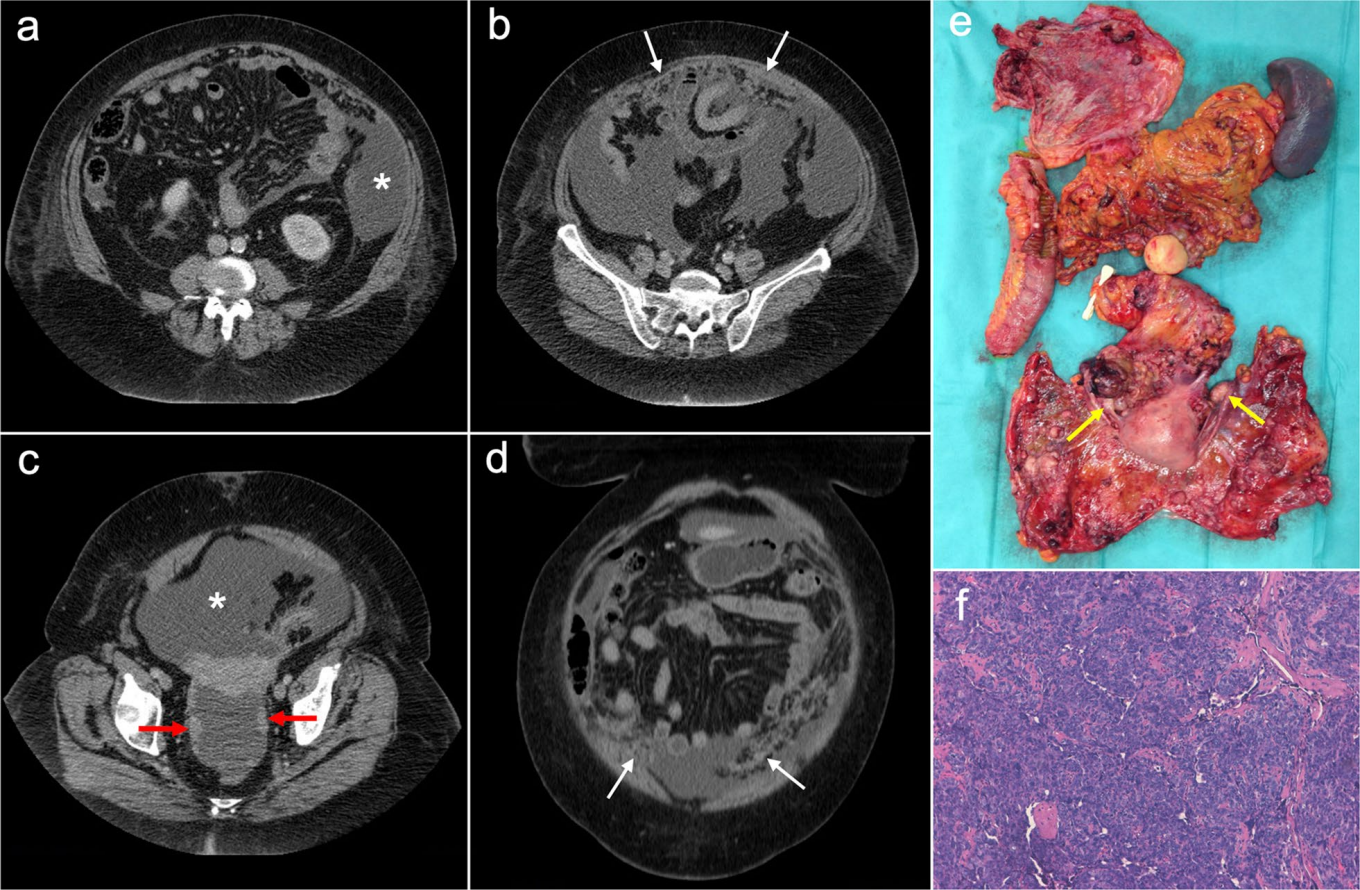
Primary peritoneal serous carcinoma in a 73-year-old woman with abdominal pain. Axial (**a–c**) and coronal (**d**) CT images in the portal phase show ascites (white asterisk), stranding of the omental fat (white arrows) and nodular thickening of the pelvic peritoneum (red arrows), with no evidence of primary gastrointestinal or ovarian tumor. **e** Surgical specimen after cytoreductive surgery shows diffuse disease in the peritoneal cavity with multiple implants, resembling ovarian carcinomatosis. Note both ovaries are normal in size and show no measurable masses (yellow arrows). **f** H&E stain photomicrograph shows a high-grade serous carcinoma with a solid pattern

Imaging features of PPSC mimic peritoneal carcinomatosis secondary to ovarian cancer, presenting with ascites, stranding of omental fat, omental nodules and masses and nodular thickening of the peritoneum, but with no detectable adnexal mass (Fig. 10). Psammomatous calcifications within nodules may be seen in up to 30% of cases [2, 31, 33]. Elevation of serum levels of CA-125 is also present in the majority of cases [2, 31].

The histology of PPSC resembles a malignant ovarian surface epithelial–stromal tumor, but ovarian involvement is absent or limited to its surface epithelium [2, 30, 31].

The treatment of choice is equivalent to ovarian carcinomatosis, with a combination of chemotherapy and CRS with or without HIPEC. The prognosis is also similar to ovarian carcinomatosis in terms of response to treatment and survival rates [34].

## Secondary tumors of the peritoneum

### Ovarian cancer

Ovarian cancer is the most common cause of death due to gynecologic malignancy, and it usually presents at an advanced stage (III or IV) due to its vague clinical symptoms, such as mild abdominal pain or malaise. Peritoneal involvement is present in approximately 70% of patients at the initial diagnosis and it is favored by the particular anatomy of the ovaries, which are not covered by the peritoneum and present a single thin layer of surface epithelium. This lack of an anatomical barrier facilitates the seeding of malignant cells into the peritoneal cavity, especially in epithelial ovarian cancers [35].

**Fig. 11:**
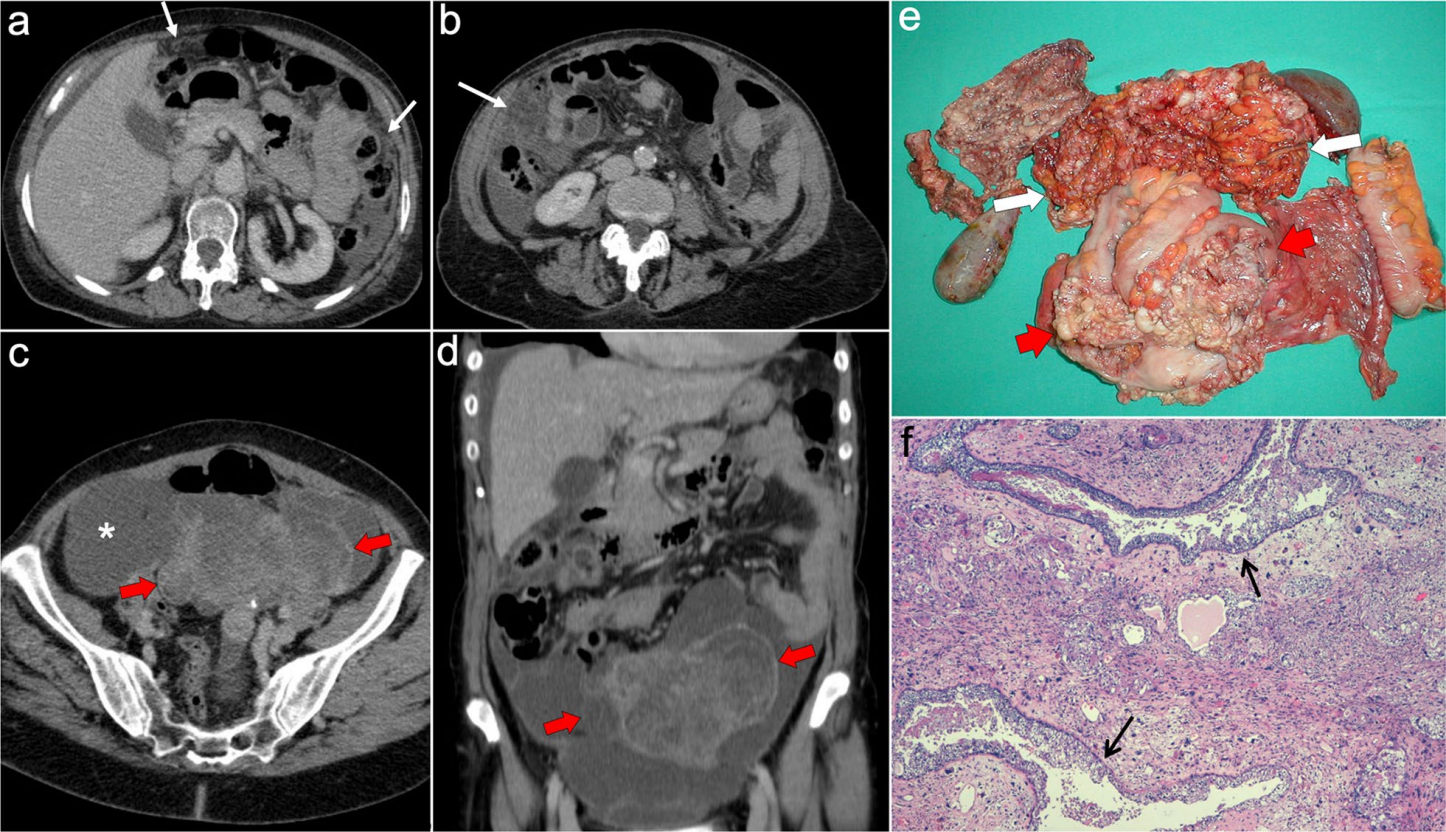
Peritoneal carcinomatosis arising from a left ovarian carcinosarcoma, in a 72-year-old woman with abdominal pain. Axial (**a–c**) and coronal (**d**) CT images in the portal phase show loculated ascites (white asterisk), stranding of the greater omentum fat (white arrows) and a heterogeneous solid-cystic mass located in the left ovarian fossa representing the primary neoplasm (red arrows). **e** Surgical specimen after cytoreductive surgery shows en bloc pelvic resection of the ovarian mass (red arrows) and omentectomy with peritoneal implants (white arrows). **f** H&E stain shows a malignant mixed epithelial tumor with papillary glands (black arrows) and sarcomatoid cells

**Fig. 12:**
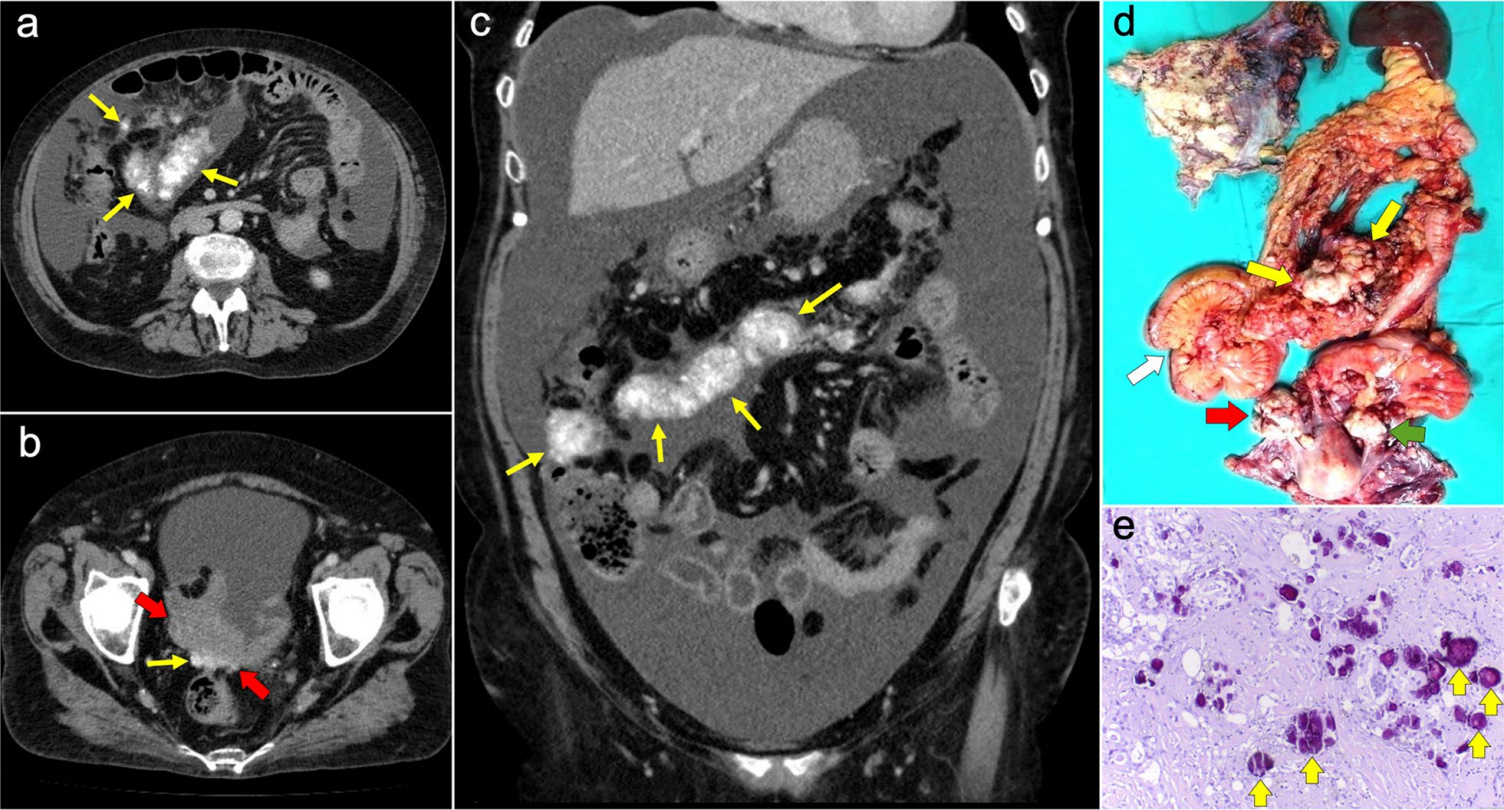
Peritoneal carcinomatosis arising from a high-grade ovarian serous adenocarcinoma, in a 77-year-old woman with abdominal distension. Axial (**a**, **b**) and coronal (**c**) CT images in the portal phase show a solid right adnexal mass (red arrows) with peripheral calcifications (yellow arrow) representing the primary tumor, which infiltrates the anterior mesorectal fascia. It shows signs of peritoneal dissemination with stranding of the mesenteric and omental fat, significant ascites and confluent solid omental and mesenteric nodules and masses. Note the presence of dystrophic calcifications within these omental and mesenteric implants, corresponding to psammoma bodies (yellow arrows). **d** Surgical specimen after cytoreductive surgery shows en bloc pelvic resection of the right ovarian mass (red arrow) with involvement of the contralateral ovary (green arrow) and the rectum, and peritoneal dissemination with infiltration of the small bowel mesentery (white arrow). Note the whitish appearance of the confluent omental masses related to the presence of dystrophic calcifications (yellow arrows). **e** H&E stain photomicrograph shows a high-grade serous adenocarcinoma with the presence of multiple psammoma bodies (yellow arrows)

Peritoneal spread secondary to ovarian cancer shows the typical appearance of peritoneal carcinomatosis on CT: ascites, stranding and nodularity of omental fat with an omental-cake appearance when it is confluent and prominent, mesenteric nodules and thickening and enhancement of peritoneal sheets (Fig. 11) [3, 35–37]. Ascites is often significant causing abdominal distension, and it may be accompanied by malignant pleural effusion [35]. Peritoneal deposits have variable appearances, from nodular to plaque-like or masses, and may contain psammomatous calcifications (Fig. 12) [35, 36]. Pelvic MRI is the technique of choice for local staging of ovarian cancer due to its superior soft tissue contrast, which allows a better depiction of the local invasion of adjacent structures [4].

Differential diagnosis must be made with PPSC, which shares similar histological and radiological features but with no evidence of adnexal mass [2]. Histology often reveals epithelial ovarian cancer, with high-grade serous carcinoma being the most common subtype [38].

The treatment of choice in stage III ovarian cancer with resectable peritoneal disease and no visceral metastases consists of a combination of CRS ± HIPEC, followed by platinum-based adjuvant chemotherapy [1, 39, 40]. In stage IV ovarian cancer or in cases with a large volume of peritoneal disease, the first option is neoadjuvant chemotherapy, which may be followed by interval CRS and adjuvant chemotherapy if there is a positive response to systemic treatment [30]. Prognosis depends on the initial stage of diagnosis. In this sense, a recent study in a cohort of 1.3 million women showed a 5-year survival rate of 26% in stage III and 14% in stage IV [41]. The volume of residual disease after CRS is considered the strongest prognostic factor for progression-free survival and overall survival [39].

### Gastrointestinal cancer

Several malignancies of the gastrointestinal tract have the potential to metastasize to the peritoneal cavity. The most frequent ones are colorectal and gastric cancers, followed by others like appendix, pancreas and gallbladder [3]. Different studies have reported that up to 5–15% of patients with colorectal cancer and 10–21% of patients with gastric cancer present with a synchronous peritoneal spread at initial diagnosis [30, 42, 43].

**Fig. 13:**
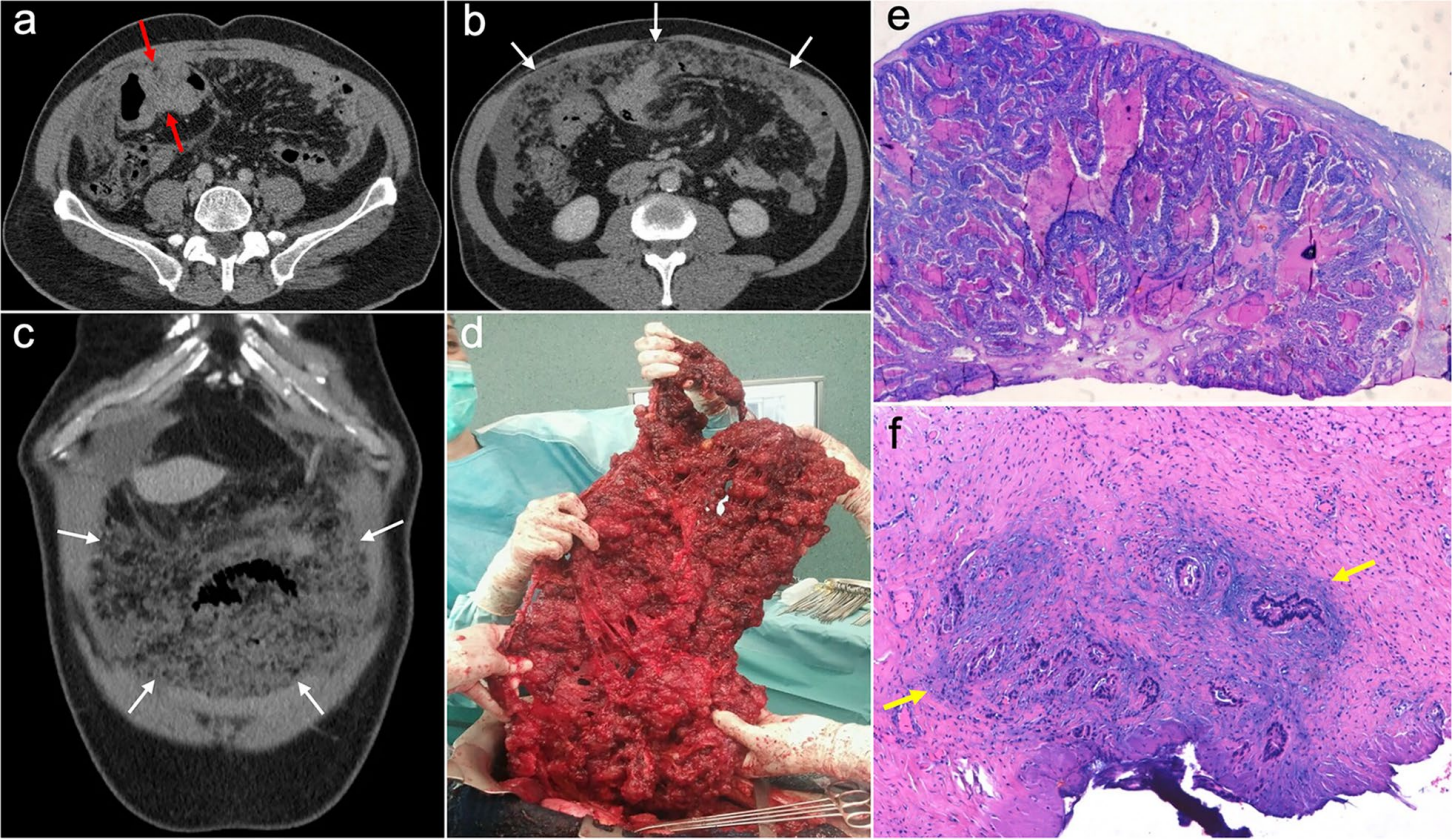
Peritoneal carcinomatosis originating from a colonic adenocarcinoma. **a** Axial CT image shows the primary neoplasm presenting as a segmental and concentric thickening of the colonic wall (red arrows). **b**, **c** Axial and coronal CT images show a typical omental-cake appearance with stranding and nodularity of the omental fat (white arrows) and mild ascites. **d** Surgical specimen of the omentectomy shows diffuse nodular infiltration of the adipose tissue that correlates with the omental-cake appearance seen on CT. **e**, **f** H&E stain photomicrographs show fibroadipose tissue with focal infiltration by moderately differentiated adenocarcinoma (yellow arrows)

**Fig. 14:**
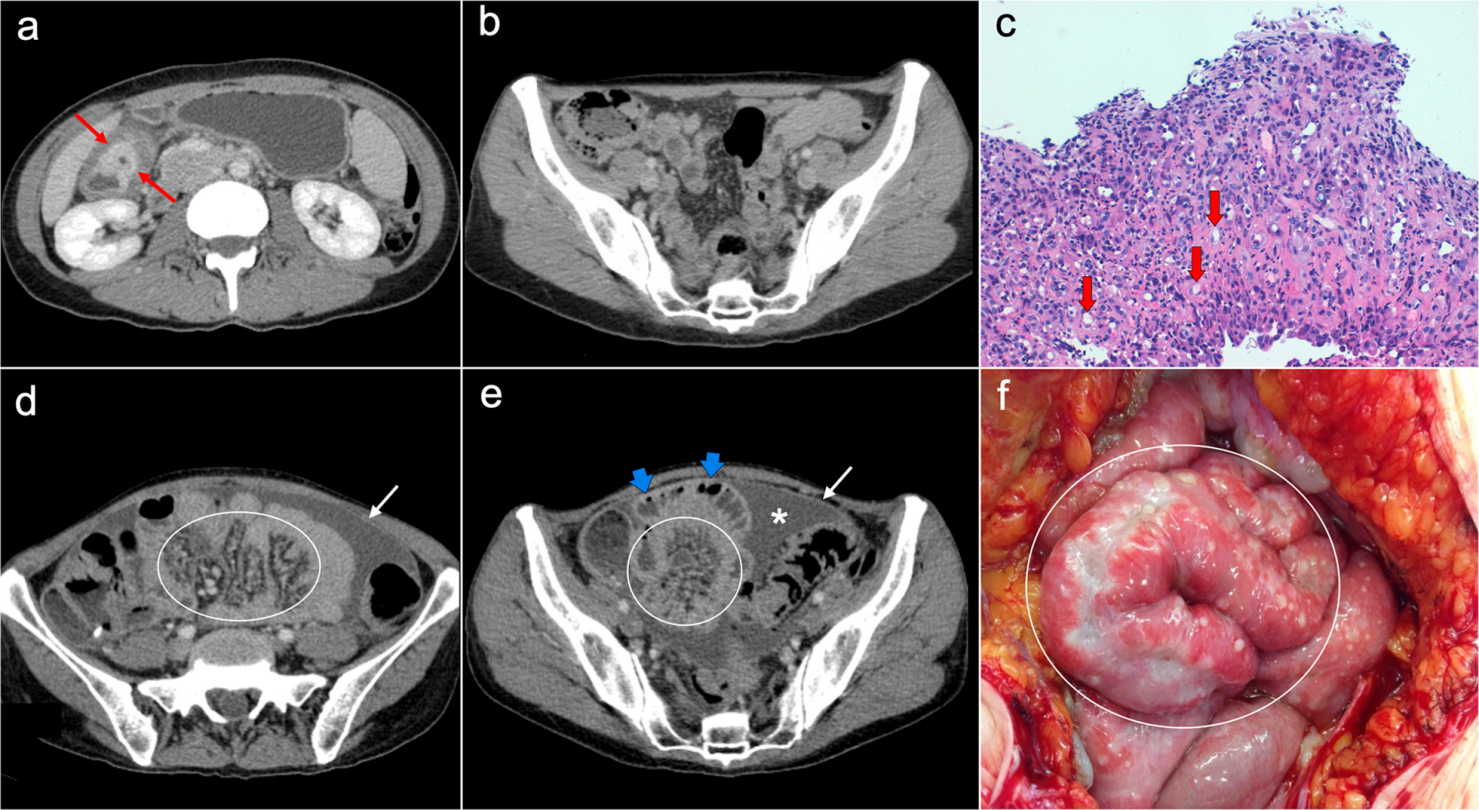
Peritoneal carcinomatosis secondary to a recurrent gastric adenocarcinoma in a 41-year-old woman. **a**, **b** Axial CT image shows the primary neoplasm as a segmental and concentric thickening of the gastric antrum (red arrows) and a normal mesenteric root, with no ascites and no signs of peritoneal dissemination. Total gastrectomy was performed and H&E stain photomicrograph (**c**) of the primary tumor showed a poorly differentiated gastric carcinoma with isolated signet ring cells (red arrows). Eleven months after surgery the patient complained of abdominal fullness, pain and constipation. Abdominal CT (**d**, **e**) was carried out showing pelvic ascites (white asterisk), peritoneal thickening (white arrows) and increased attenuation of the mesenteric root fat suggestive of “stellate mesentery” (white oval in **d**, white circle in **e**) with distension of adjacent ileal loops (blue arrows), findings that raised the suspicion of peritoneal recurrence. Initial laparoscopy did not allow an adequate visualization of the mesenteric root and ascitic fluid cytology was negative for malignancy, so eventually exploratory laparotomy was performed to confirm the presence of peritoneal disease. Intraoperative view (**f**) showed fixation and tethering of the ileal loops secondary to confluent plaque-like whitish lesions in the small bowel serosa (white circle), representing extensive tumoral involvement with retraction of the adjacent mesentery

Imaging features are similar to those previously described on ovarian cancer, and relate to the classic appearance of peritoneal carcinomatosis: ascites, stranding of omental fat, omental nodules and masses, mesenteric nodules and thickening and enhancement of peritoneal sheets (Fig. 13). In advanced stages, peritoneal metastases commonly spread to the mesentery with a “stellate appearance,” characterized by increased attenuation of the mesenteric fat and perivascular soft tissue thickening caused by microscopic infiltration of adipose tissue along the mesenteric blood vessels (Fig. 14) [3, 5, 44]. It is often associated with fixation, tethering and distortion of small bowel loops, with loss of fat planes and eventually bowel obstruction [3].

**Fig. 15:**
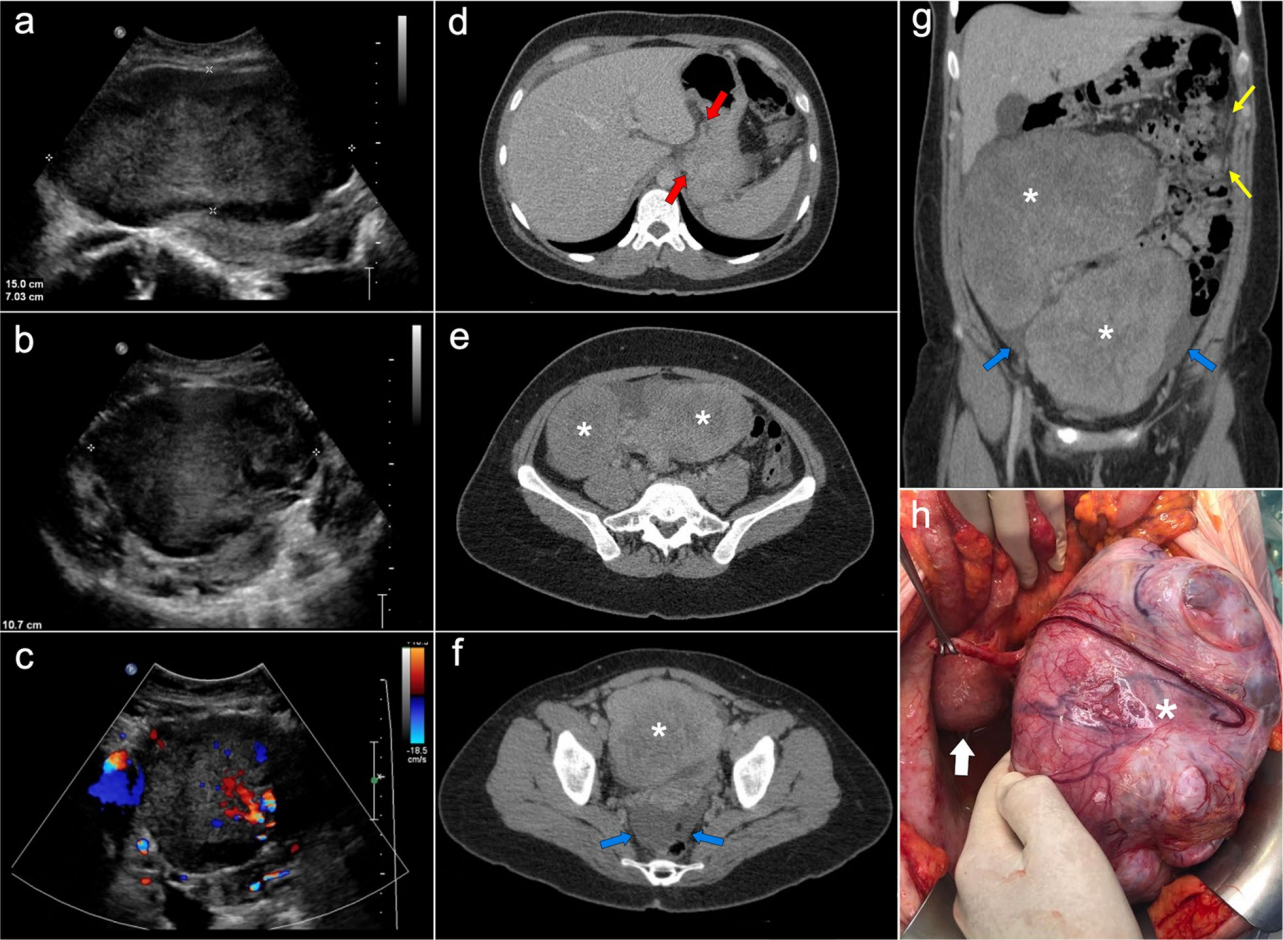
Krukenberg tumors and peritoneal carcinomatosis secondary to a diffuse gastric adenocarcinoma with signet ring cells, in a 32-year-old woman with non-specific abdominal discomfort. First, an abdominal ultrasound was performed, which showed two voluminous solid masses in the lower half of the abdomen (**a**, **b**) and demonstrated vascularity within them by color Doppler (**c**). Axial (**d–f**) and coronal (**g**) CT images with intravenous contrast in the portal phase confirmed the presence of two large solid masses of probable adnexal origin (white asterisks), associated with ascitic fluid (blue arrows) and stranding of the omental fat (yellow arrows) suspicious of peritoneal carcinomatosis. Note also the presence of an ill-defined thickening of the gastric wall in the subcardial region, with extension toward the gastrohepatic ligament, suggestive of a primary gastric malignancy (**d**, red arrows). Subsequently, gastroscopy with biopsy was performed, confirming the diagnosis of a gastric adenocarcinoma with signet ring cells. Palliative debulking surgery with bilateral oophorectomy was decided after multidisciplinary discussion of the case. **h** Intraoperative view of the pelvis shows the left adnexal mass (white asterisk) and the inferior pole of the right adnexal mass (white arrow), which extends superiorly toward the upper abdomen

Primary and recurrent gastric cancers tend to present miliary dissemination, with tiny nodules difficult to detect on CT, PET/CT or MRI. In this case, it is important to look for indirect signs of peritoneal dissemination, like ascites or subtle stranding of the mesenteric fat [45]. Both colorectal and gastric cancers may produce ovarian metastases known as Krukenberg tumors, which must be differentiated from primary ovarian carcinomas (Fig. 15) [46].

**Fig. 16:**
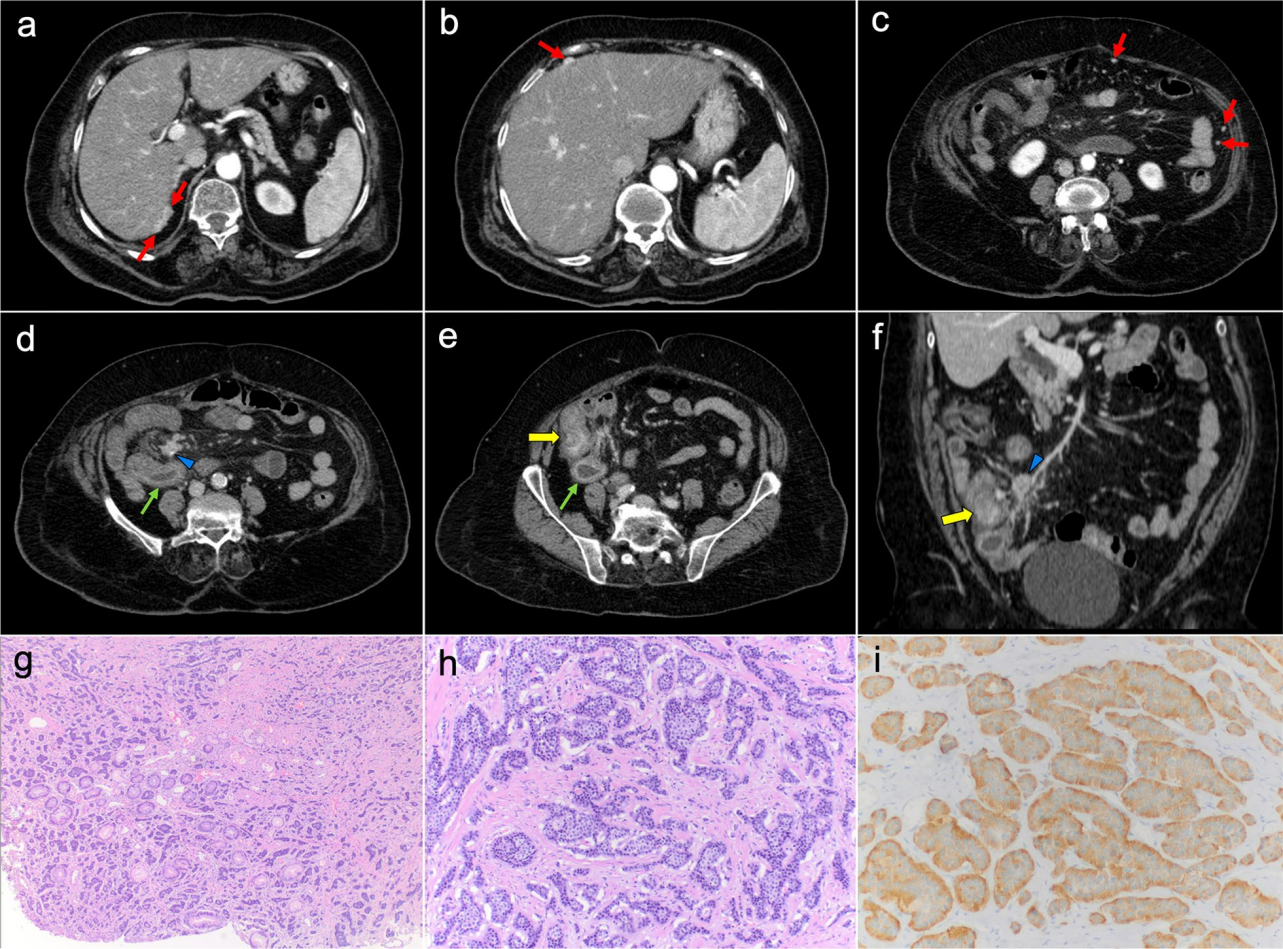
Gastrointestinal NET of the distal ileum with peritoneal dissemination in a 73-year-old woman, who had been complaining of diarrhea and symptoms of intestinal subocclusion for several months. **a–c** Axial CT images with intravenous contrast in the arterial phase show multiple hypervascular peritoneal implants of small size in the perihepatic space and the omental fat (red arrows). Axial (**d**, **e**) and coronal (**f**) CT images with intravenous contrast in the portal phase show an enhancing pseudonodular lesion in the distal ileum (yellow arrows), representing a primary NET. The tumor invades directly the adjacent mesentery, forming an enhancing soft tissue mass with punctate calcification (blue arrowhead). This mass retracts the mesenteric vessels, producing mild distension and mural thickening of the ileal loops in relation to chronic ischemic ileitis (green arrows). **g**, **h** H&E photomicrographs show a fibrous stroma with monomorphic cells that arrange in small groups and gland like structures, presenting fairly uniform nuclei, coarsely stippled chromatin ("salt and pepper") and finely granular cytoplasm, corresponding to a well-differentiated neuroendocrine tumor. **i** Immunohistochemistry shows positive staining for synaptophysin, confirming the neuroendocrine origin

When gastrointestinal tumors and their peritoneal deposits show a hypervascular behavior on CT and MRI, with early contrast uptake in the arterial phase, they should raise the suspicion of a neuroendocrine origin [4]. These neuroendocrine tumors (NETs) are a distinct group of small and slow-growing neoplasms that may present with peritoneal dissemination in 10–33% of cases, often associated with synchronous lymph nodes and liver metastases [47, 48]. When peritoneal spread is present, the most common reported location of the primary tumor is the ileum or the appendix [48, 49]. Appendicular NET is often difficult to identify on CT and MRI because of its small size [50]. On the other hand, ileal NET shows typical imaging findings due to invasion of the adjacent mesentery. It presents as a mesenteric soft tissue mass with frequent calcifications and spiculated margins due to desmoplastic reaction, which retracts mesenteric vessels producing chronic ischemic ileitis with mural thickening, tethering and distension of ileal loops (Fig. 16) [51]. Staging of advanced NETs benefits from the use of functional imaging techniques such as [111In]-octreotide scintigraphy, [68 Ga] somatostatin analog PET/CT and [18F] FDG PET/CT, which improve the detection of peritoneal and visceral metastases [50, 52].

Different pathologic cell lines may cause peritoneal carcinomatosis of gastrointestinal origin. Adenocarcinoma is the most frequent histological type, and it is not unusual the presence of signet ring cells [3]. Immunohistochemistry is essential, as most gastrointestinal tumors can be differentiated by their unique immunohistochemical profile [53]. In gastrointestinal NETs, immunohistochemical markers like chromogranin A, synaptophysin, p53, retinoblastoma protein (Rb) and Ki-67 proliferation index are also essential for diagnosis, grading and prognosis, allowing differentiation between well-differentiated neuroendocrine tumors (previously known as carcinoid tumors) and poorly differentiated neuroendocrine carcinomas [54, 55].

The treatment and prognosis of gastrointestinal cancers with peritoneal spread are variable and are conditioned by the histology of the primary tumor, the absence of extraperitoneal disease and the capacity to achieve a complete cytoreduction during surgery [42]. In patients with colorectal cancer limited to the peritoneum, CRS ± HIPEC is the treatment of choice when an optimal surgery is feasible (even in selected patients with liver metastases) and long-term survival has been demonstrated in different studies, with a 5-year survival rate of up to 45% [42]. Conversely, patients with peritoneal metastases of gastric or pancreaticobiliary origin show poor prognosis and significantly lower survival rates in comparison with colorectal carcinomatosis, with a median survival of 4–5 months for gastric carcinomatosis and only 1–2 months for pancreatic carcinomatosis. In this setting, chemotherapy is the treatment of choice, and interval CRS ± HIPEC may only be considered in highly selected patients with low tumor burden (PCI < 10), no evidence of visceral metastases and a positive response to previous systemic treatment [30].

### Pseudomyxoma peritonei

Pseudomyxoma peritonei (PMP) is a clinicopathological entity defined by the persistent accumulation of mucin inside the peritoneal cavity secondary to a mucinous tumor. This material is redistributed along the peritoneal cavity following the normal flow of peritoneal fluid [56–58]. The most frequent origin of PMP is a perforated appendiceal mucinous neoplasm (AMN), which depending on the grade of cytologic atypia can be subclassified as a low-grade (LAMN) or a high-grade (HAMN) mucinous neoplasm [56–58]. Another frequent origin of PMP is a mucinous adenocarcinoma of the colon or appendix, characterized by infiltrative invasion and frequent presence of signet ring cells. Much less commonly PMP can be secondary to other mucinous tumors of ovarian, gastric, pancreatic or urachal origin.

Macroscopically, PMP is defined by the presence of yellowish gelatinous material within the abdominal cavity, covering the peritoneal surface. Microscopically, recent updates have been made to the original classification by Ronnett et al. [59] in order to avoid confusion with the diagnostic terminology. In this sense, consensus guidelines from the Peritoneal Surface Oncology Group (PSOGI) and other panels of experts have classified PMP into three basic groups [57, 58, 60]:

- *PMP with low-grade histological features* Equivalent to the old term of disseminated peritoneal adenomucinosis (DPAM). This variant almost always arises from a LAMN and is characterized by abundant lakes of mucin containing scanty strips of benign-appearing epithelial cells with slight nuclear atypia and occasional mitosis. It has a much better prognosis than the two other groups, with a reported 5-year survival rate of around 90–100% [60, 61].
- *PMP with high-grade histological features* Equivalent to the old term of peritoneal mucinous carcinomatosis (PMCA). It is generally produced by a HAMN or a mucinous adenocarcinoma and consists of mucin pools with abundant cellularity, high-grade cytologic atypia, numerous mitosis and infiltrative invasion. It has a reported 5-year survival rate of around 40–60% [60, 61].
- *PMP with signet ring cells* Similar to a high-grade PMP but with the presence of signet ring cells, which correlates with a more aggressive behavior and a reported 5-year survival rate around 20% [60, 61].

**Fig. 17:**
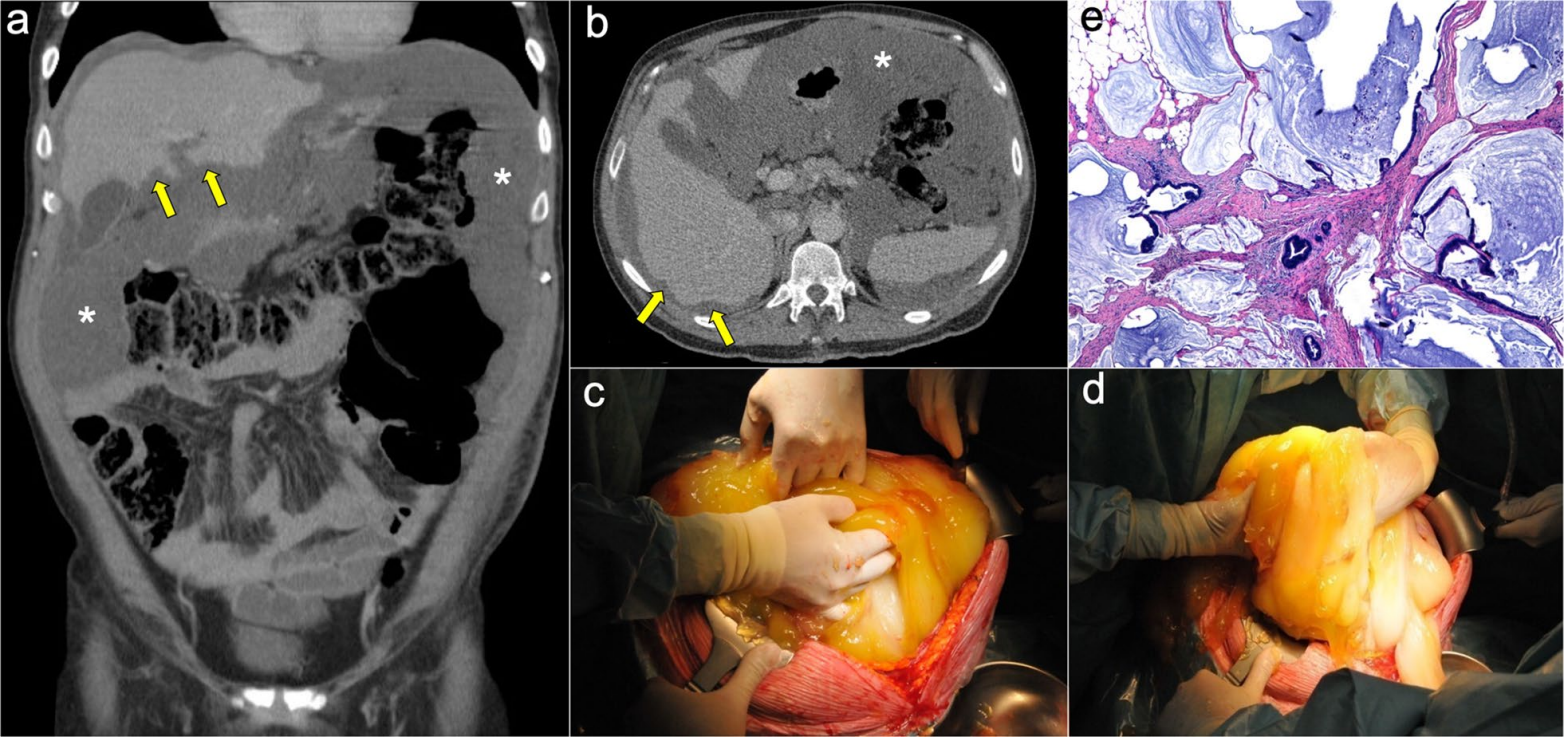
Pseudomyxoma peritonei arising from a ruptured appendiceal mucinous neoplasm. Coronal (**a**) and axial (**b**) CT images in the portal phase show voluminous mucinous ascites (white asterisks) with a diffuse distribution. Note the loculated aspect of the ascites with scalloping of the liver surface (yellow arrows). In this case the primary appendiceal neoplasm was difficult to identify on CT due to its massive rupture. **c**, **d** Intraoperative views show accumulation of yellowish gelatinous material within the abdominal cavity. **e** H&E stain photomicrograph shows large bluish lakes of mucin with strips of epithelial cells that show minimal cytologic atypia, corresponding to a LAMN

**Fig. 18:**
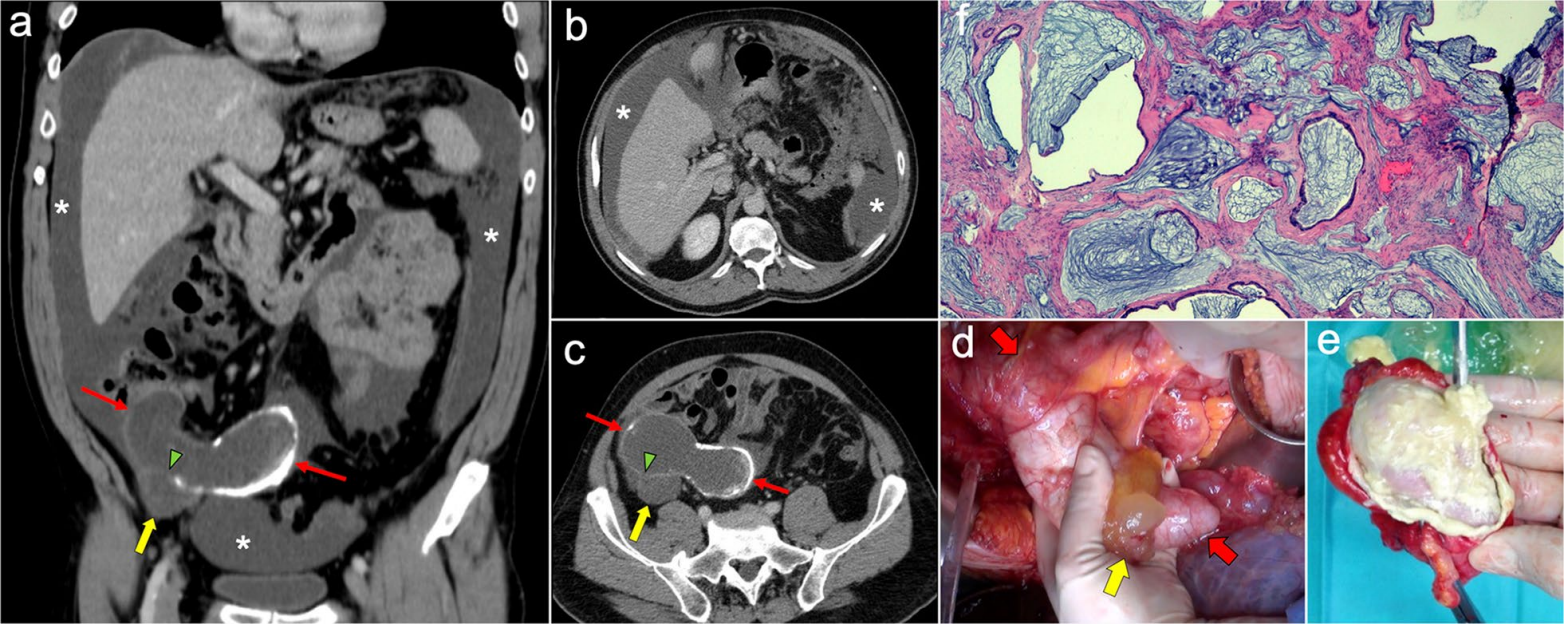
Pseudomyxoma peritonei arising from a perforated appendiceal mucinous neoplasm, in a 65-year-old man complaining of abdominal fullness and discomfort. Coronal (**a**) and axial (**b**, **c**) CT images in the portal phase show a cystic dilatation of the appendix with thin internal septa and peripheral calcifications, representing an appendiceal mucinous neoplasm (red arrows). Note the focal perforation of the tumor (green arrowhead) with mucin leakage (yellow arrows), as well as the loculated ascites representing acellular mucin pools (white asterisks). **d** Intraoperative view shows the perforated appendiceal mucinous neoplasm (red arrows) with output of yellowish mucinous material (yellow arrow). **e** Dissection of the surgical specimen of appendectomy, showing the presence of whitish calcifications in the appendiceal wall. **f** H&E stain photomicrograph shows bluish lakes of mucin with sheets of epithelial cells and minimal atypia, findings consistent with a LAMN

**Fig. 19:**
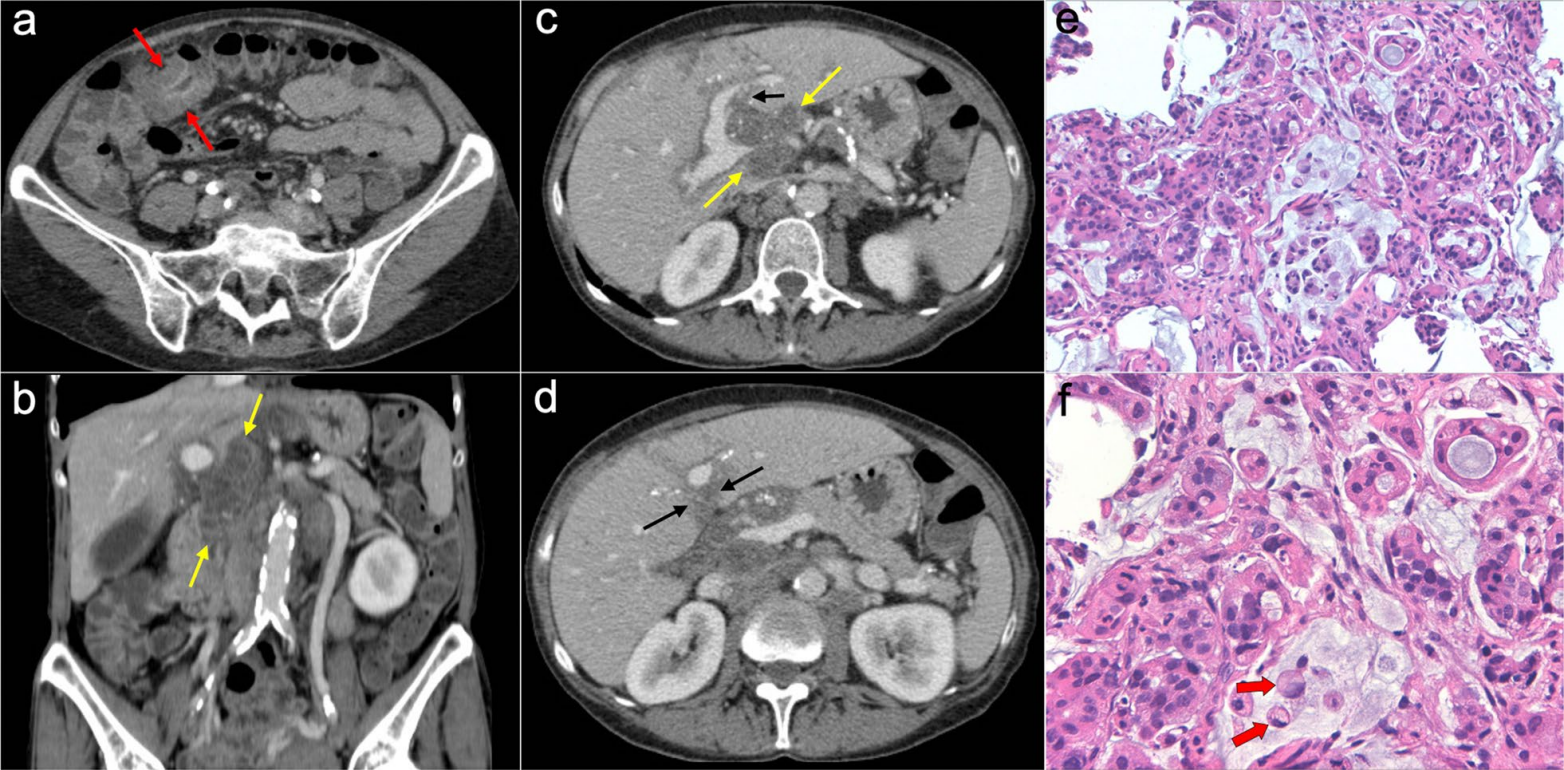
Pseudomyxoma peritonei with signet ring cells arising from a mucinous adenocarcinoma of the colon. **a** Axial CT image in the portal phase shows the primary neoplasm presenting as segmental and concentric thickening of the ascending colon (red arrows). Coronal (**b**) and axial (**c**, **d**) CT images show significant disease in the epigastric region (zone 2), with hypodense infiltration of the hepatic hilum (yellow arrows) associated with small calcifications and severe stenosis of the porta hepatis and inferior vena cava. Extension through the intrahepatic periportal space and the falciform ligament is also present (black arrows). Note the more aggressive appearance in comparison with the classic pseudomyxoma secondary to a LAMN. **e**, **f**) H&E stain photomicrographs show a high-grade mucinous adenocarcinoma with signet ring cells (red arrows)

**Fig. 20:**
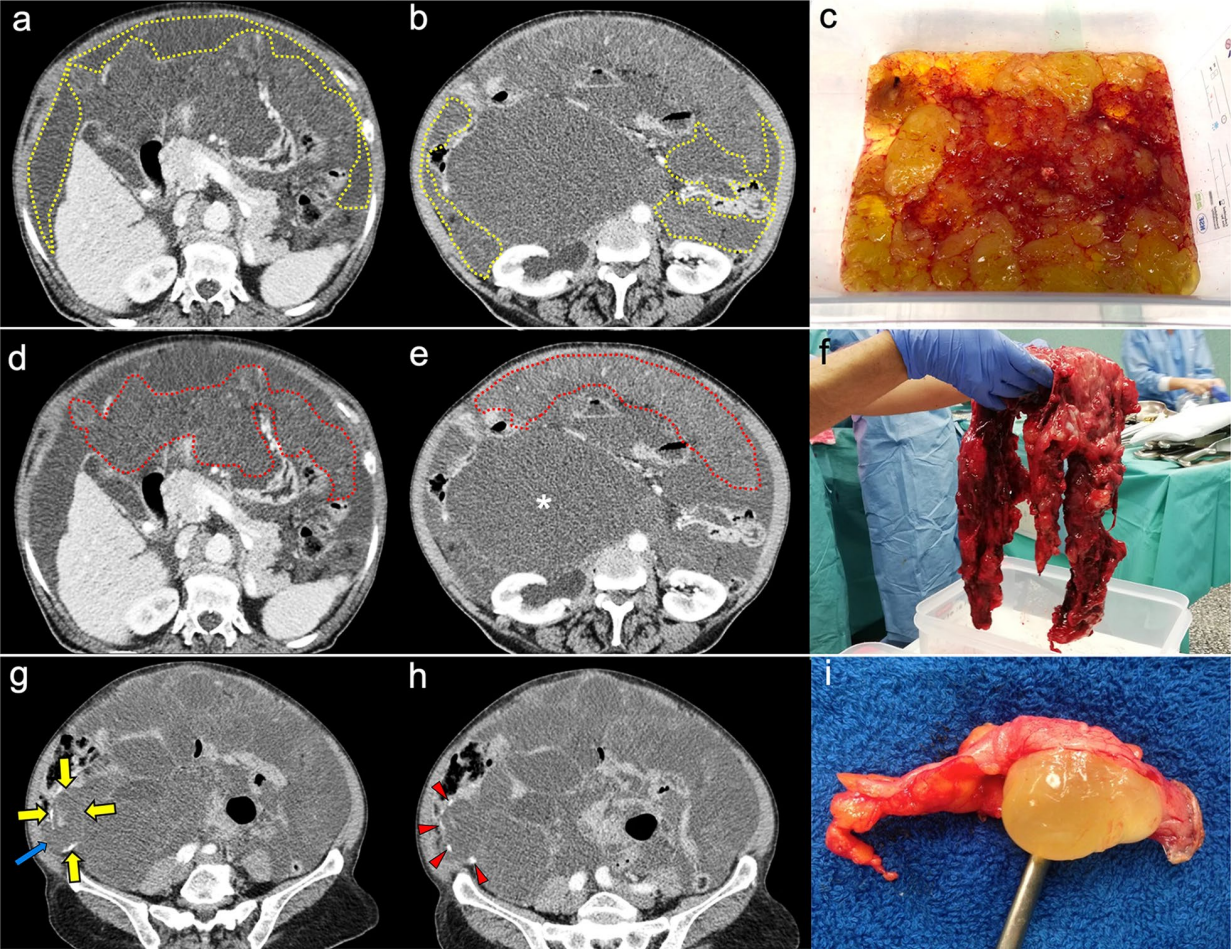
High-volume pseudomyxoma originating from a ruptured appendiceal mucinous neoplasm, with coexistence of low-grade and high-grade histologic features. **a**, **b**) Axial CT images in the portal phase show high volume of disease, with homogenous loculated ascites representing acellular mucin pools (yellow dots). **c** Surgical correlation of acellular mucin pools, appearing as yellowish gelatinous material. **d**, **e** Same axial CT images in the portal phase show coexistence of hypodense acellular mucin with dense omental disease (red dots), representing cellular areas of mucinous carcinomatosis. **f** Surgical correlation of mucinous carcinomatosis, appearing as fleshy reddish solid material. **g**, **h** Axial CT images in the portal phase at a lower level show a hypodense tubular image in the right iliac fossa (yellow arrows) with calcified walls (red arrowheads), representing an appendiceal mucinous neoplasm. Perforation can also be seen in its right lateral wall (blue arrow). **i** Appendectomy specimen shows a perforated mucinous neoplasm with output of yellowish gelatinous material

The main imaging feature of PMP on CT is a high-volume loculated mucinous ascites causing mass effect on solid abdominal organs like the liver or the spleen, which develop a characteristic scalloped appearance (Fig. 17). The appendix should always be scrutinized looking for a primary AMN, that presents as a dilated appendix with hypodense material, frequent septa, thin walls and peripheral calcifications (Fig. 18) [3, 5, 50, 62, 63]. Peritoneal mucinous deposits may also present linear or punctate calcifications [50]. A more infiltrative behavior of mucinous implants is seen in cases with high-grade histological features and signet ring cells, with frequent vascular involvement of the epigastric region (Fig. 19). Ovarian involvement in the form of hypodense mucinous implants is also frequent, and must not be misdiagnosed with a primary ovarian neoplasm [50]. Solid peritoneal implants and omental cake may also be seen, more often on PMP with high-grade histological features and signet ring cells, but they can also be present in patients with low-grade histological features [55]. In this sense, it is not unusual to find in the same abdominal CT scan signs of low-grade disease, such as hypodense loculated ascites representing acellular mucin pools, coexisting with high-grade features like dense areas of infiltrative appearance representing mucinous carcinomatosis (Fig. 20).

**Fig. 21:**
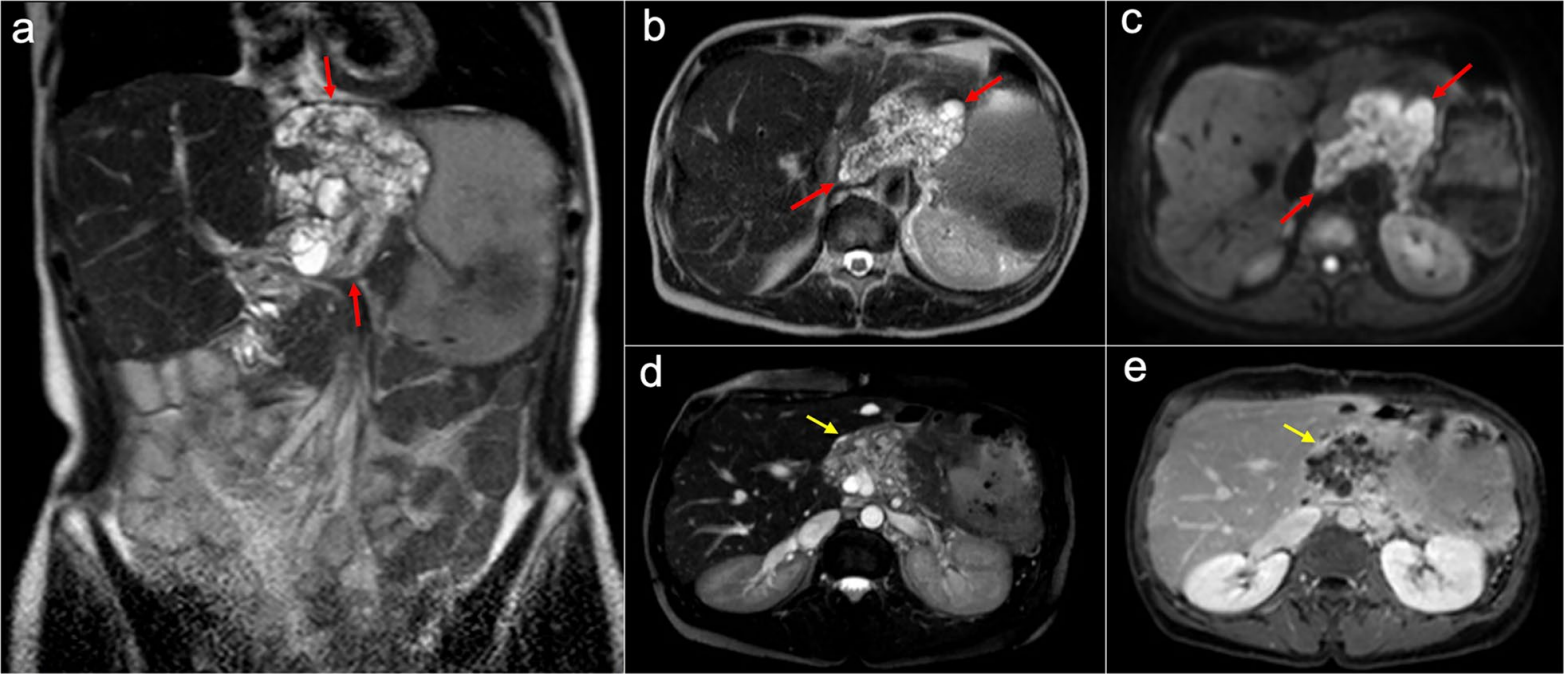
Peritoneal MRI depicting recurrence of a pseudomyxoma peritonei with high-grade histologic features, secondary to a perforated appendiceal mucinous neoplasm. Coronal (**a**) and axial (**b**) T2-weighted images show abundant hyperintense and multiseptated mucinous disease in the lesser sac (red arrows), that shows wide contact with the left suprahepatic vein, inferior vena cava, lesser gastric curvature and pancreas. **c** Diffusion-weighted image with intermediate b-value (b = 500) shows hyperintensity of the mucinous material (red arrows). Axial balanced turbo field echo sequence (**d**) and axial T1-weighted imaged with fat saturation after intravenous administration of gadolinium in the portal phase (**e**) show extension of mucinous disease toward the round ligament, with severe stenosis of left portal intrahepatic vein (yellow arrows) and peripheral and septal enhancement

Although CT remains the most commonly used imaging technique to depict PMP, peritoneal MRI has also been reported as a promising tool for preoperative staging and surveillance. This modality presents high sensitivity in the detection of mucinous implants, which appear as hyperintense cystic-like lesions with internal septa on T2-weighted images. On diffusion-weighted imaging, these mucinous lesions are best depicted using intermediate b-values of 400–500 s/mm^2^ and usually show hyperintensity with high ADC values (although lower ADC values may be seen in cases with high-grade histological features and signet ring cells) (Fig. 21) [14, 15, 64]. PET/CT is not recommended because of the low metabolic activity of mucinous neoplasms, although it may be useful for the detection of systemic metastases in aggressive variants [56].

In PMP with low-grade histological features, CRS with HIPEC is the treatment of choice, with an excellent prognosis. Despite frequent recurrences, this therapeutic approach is also feasible in selected cases of high-grade PMP with or without signet ring cells, sometimes associated with adjuvant chemotherapy [58, 61].

### Sarcomas

Sarcomas are a heterogeneous group of malignant neoplasms that may occur in many different anatomical sites. The spreading of sarcomas through the peritoneum is known as peritoneal sarcomatosis [3, 30]. The most frequent origin of peritoneal sarcomatosis is malignant gastrointestinal stromal tumors (GISTs), which commonly spread directly to the peritoneal cavity by extension of the tumor through the serosal surface of the bowel or stomach [3, 65]. Other sarcomas with frequent peritoneal extension are leiomyosarcomas and liposarcomas. In soft tissue and extremity sarcomas peritoneal spread is unusual, and it is presumed to occur through a hematogenous route [3].

**Fig. 22:**
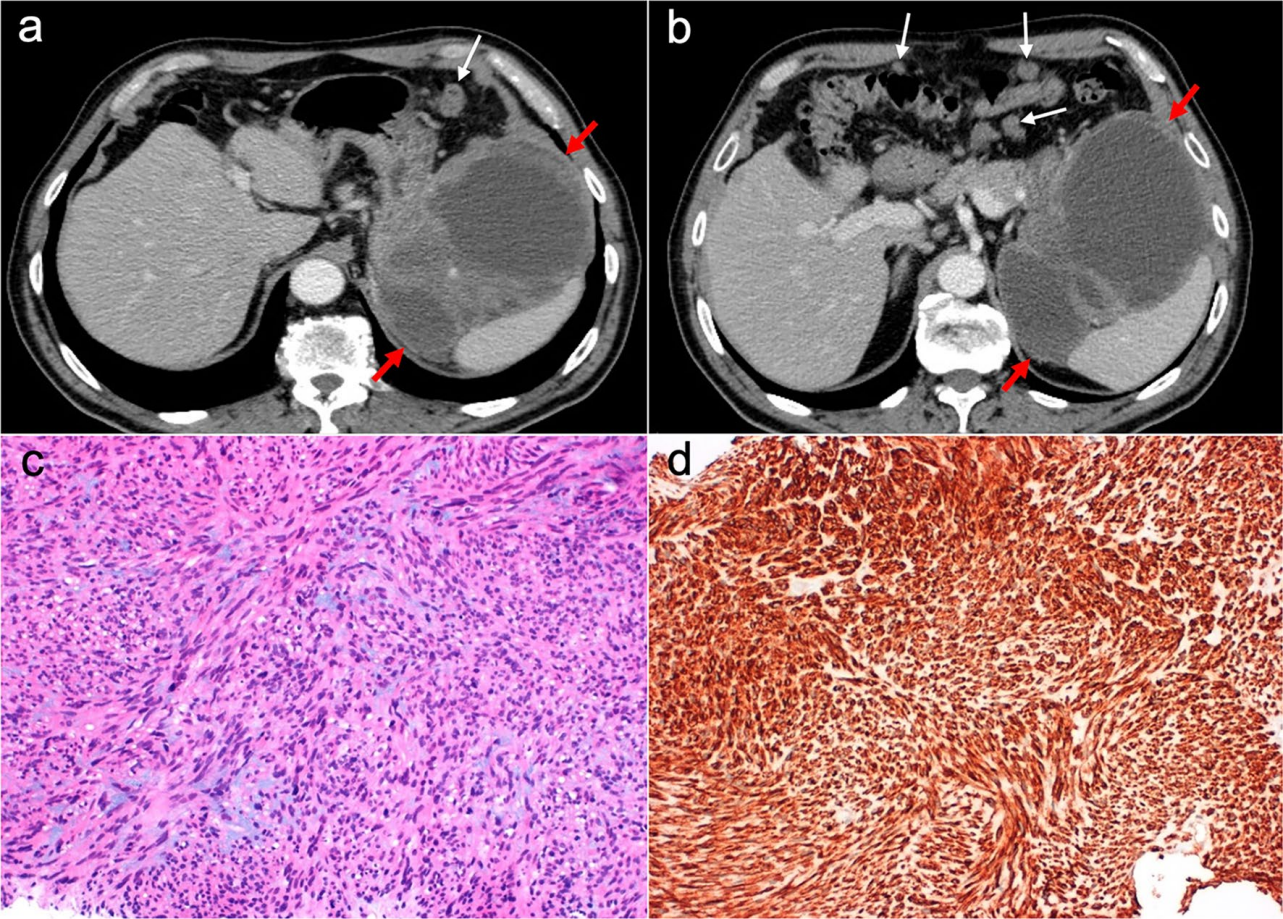
Peritoneal sarcomatosis arising from a GIST of the stomach. **a**, **b**) Axial CT images in the portal phase show a large necrotic and exophytic mass arising from the greater curvature of the stomach (red arrows), with solid omental nodules corresponding to peritoneal implants (white arrows). **c** H&E stain photomicrograph shows a cellular tumor with a storiform pattern and moderate cytologic atypia. **d** Immunohistochemistry shows positive staining for c-Kit, confirming the diagnosis

**Fig. 23:**
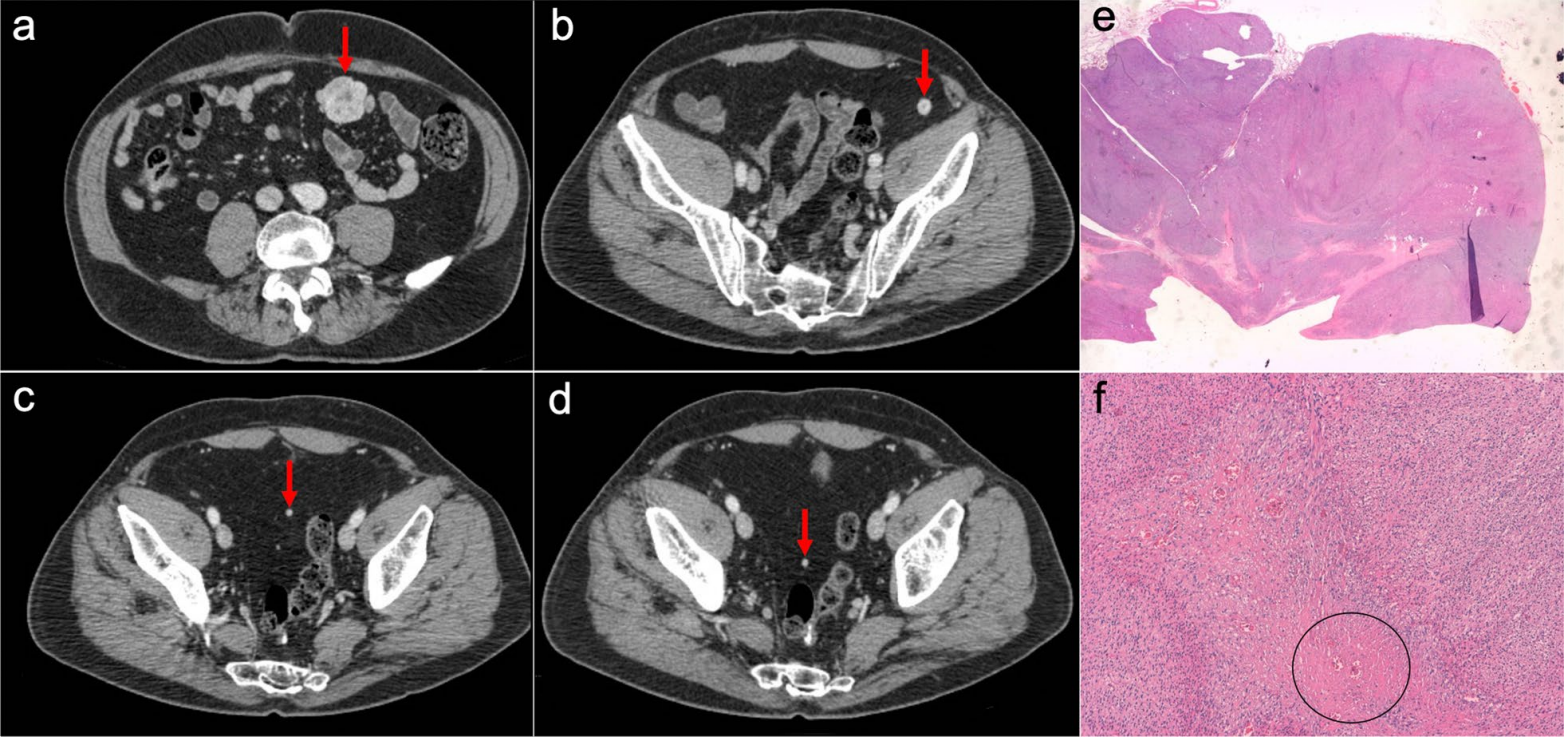
Peritoneal sarcomatosis secondary to recurrent leiomyosarcoma of the rectum. **a–d** Axial CT images in the portal phase show hypervascular solid omental and mesenteric nodules with a random distribution, corresponding to peritoneal implants (red arrows). **e**, **f** H&E stain photomicrographs show a cellular tumor with spindle-shaped cells, necrosis (circle) and moderate cytologic atypia

Imaging features on CT and MRI consist of solid omental and mesenteric nodular masses with random distribution, mimicking peritoneal carcinomatosis (Figs. 22, 23). However, it should be noted that in sarcomatosis ascites is usually minimal or absent, and peritoneal implants more often show a spherical morphology, with frequent hypervascularity and bulky heterogeneous masses [65, 66]. The primary tumor usually presents as a large mass with heterogeneous contrast uptake and necrotic areas. In the particular case of GISTs, they present as well-circumscribed tumors arising from the stomach or small bowel wall, with an extraluminal growth and a necrotic center. Liver metastases are frequent and there is no evidence of lymph node enlargement [66]. The latter feature may be useful to distinguish them from gastrointestinal NETs, which share similar hypervascular behavior but frequently manifest with regional node involvement [50, 51].

**Fig. 24:**
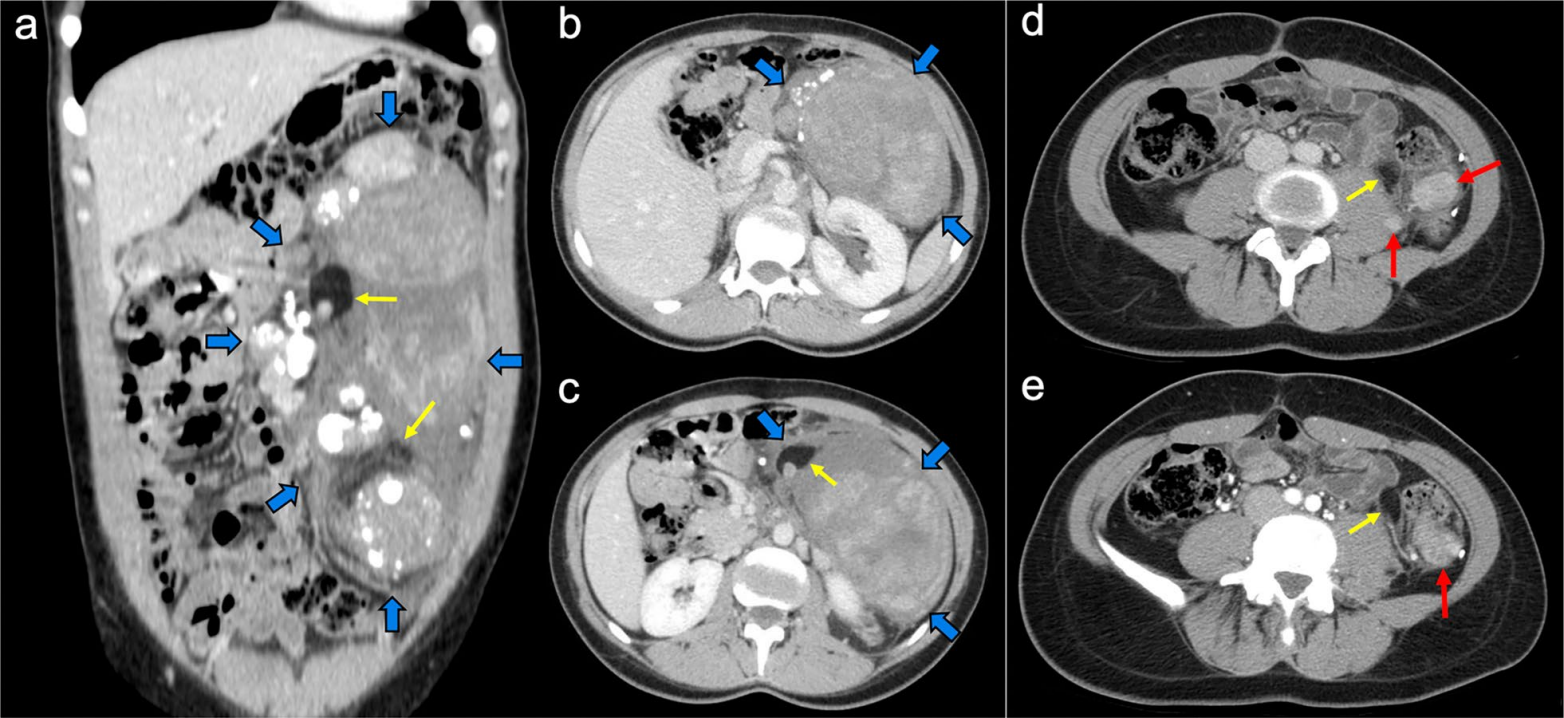
Retroperitoneal liposarcoma with peritoneal and retroperitoneal recurrence in a 32-year-old woman. Coronal (**a**) and axial (**b**, **c**) CT images in the portal phase show a voluminous solid mass with calcifications arising from the anterior perirenal fat (blue arrows) and containing macroscopic fat (yellow arrows). Complete surgical resection was made and the pathological specimen showed a mixture of well-differentiated and dedifferentiated areas of LPS. **d**, **e** Follow-up CT performed 20 months after surgery showed multifocal peritoneal and retroperitoneal recurrence consisting of a combination of dedifferentiated solid nodules (red arrows) and well-differentiated fatty implants (yellow arrows), located mainly in the left paracolic gutter and the lateral margin of the psoas muscle

The presence of macroscopic fat within the primary tumor or within peritoneal implants should raise the suspicion of liposarcoma (LPS), which is most commonly located in the retroperitoneum and is usually composed of varying proportions of adipose tissue, thick septa and nodular soft tissue elements. Adipose tissue predominates in well-differentiated LPS, whereas soft tissue nodules and masses with less lipomatous component are characteristic of aggressive histologic subtypes such as dedifferentiated LPS and pleomorphic LPS. The latter tumors also have a higher risk of peritoneal dissemination and local recurrence. It must be taken into account that different histological subtypes may coexist in the same tumor (Fig. 24) [67–69].

From a histological perspective, both GISTs and leiomyosarcomas share similar features, so a definitive diagnosis relies on immunohistochemical data: a vast majority of GISTs express c-KIT protein (CD117) and CD34, whereas leiomyosarcomas often express desmin and smooth muscle actin [70, 71]. The LPS is generally composed of a mixture of variable-sized mature adipocytes and lipoblasts, with focal nuclear atypia and hyperchromatic stromal spindle cells [68].

The treatment of choice in most cases of peritoneal sarcomatosis is based on a combination of chemotherapy or tyrosine kinase inhibitors (such as imatinib on GISTs) and an optimal CRS (when feasible) [71]. In LPS, surgical excision is the treatment of choice and the use of neoadjuvant or adjuvant chemotherapy or radiotherapy is limited to large high-grade LPS [67].

### Lymphoma

Peritoneal spread of lymphomas is rare and is known as peritoneal lymphomatosis. The most frequent origin is a preexisting non-Hodgkin B-cell Lymphoma [72–76]. Primary lymphoma of the peritoneum without visceral involvement is known as primary effusion lymphoma, and it is a rare entity found in immunocompromised patients, almost exclusively in patients with human immunodeficiency virus [72, 73].

**Fig. 25:**
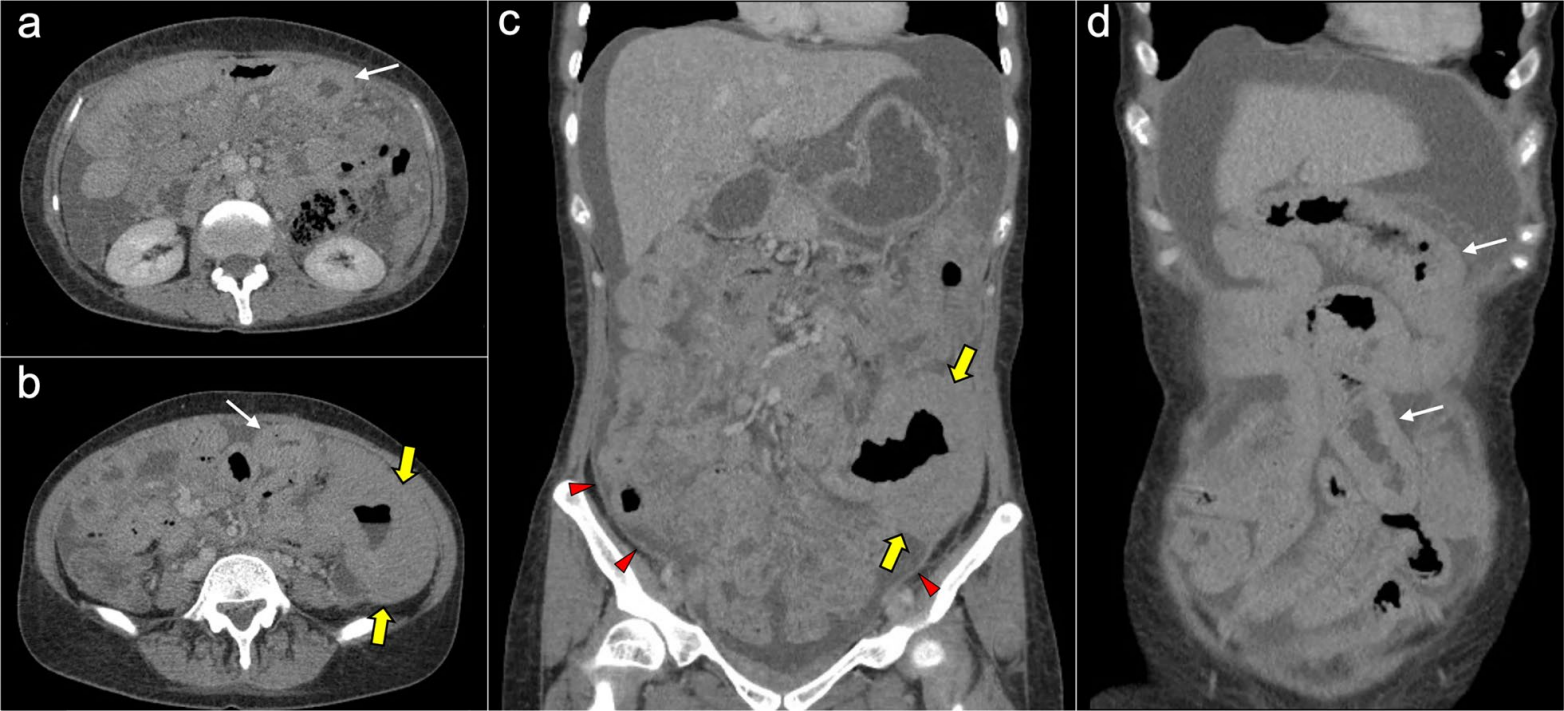
Peritoneal lymphomatosis in a 37-year-old woman with abdominal distension and loss of weight. Axial (**a**, **b**) and coronal (**c**, **d**) CT images in the portal phase show ascites, stranding of the mesenteric fat, diffuse thickening of the peritoneal folds (red arrowheads) and wall thickening of small bowel loops (white arrows). Note the presence of a mildly enhancing bulky mass in the left paracolic gutter with segmental encasement of the ascending colon (yellow arrows). The final diagnosis of B-cell non-Hodgkin's lymphoma was confirmed after ultrasound-guided biopsy of the bulky mass and cytology of the ascitic fluid

**Fig. 26:**
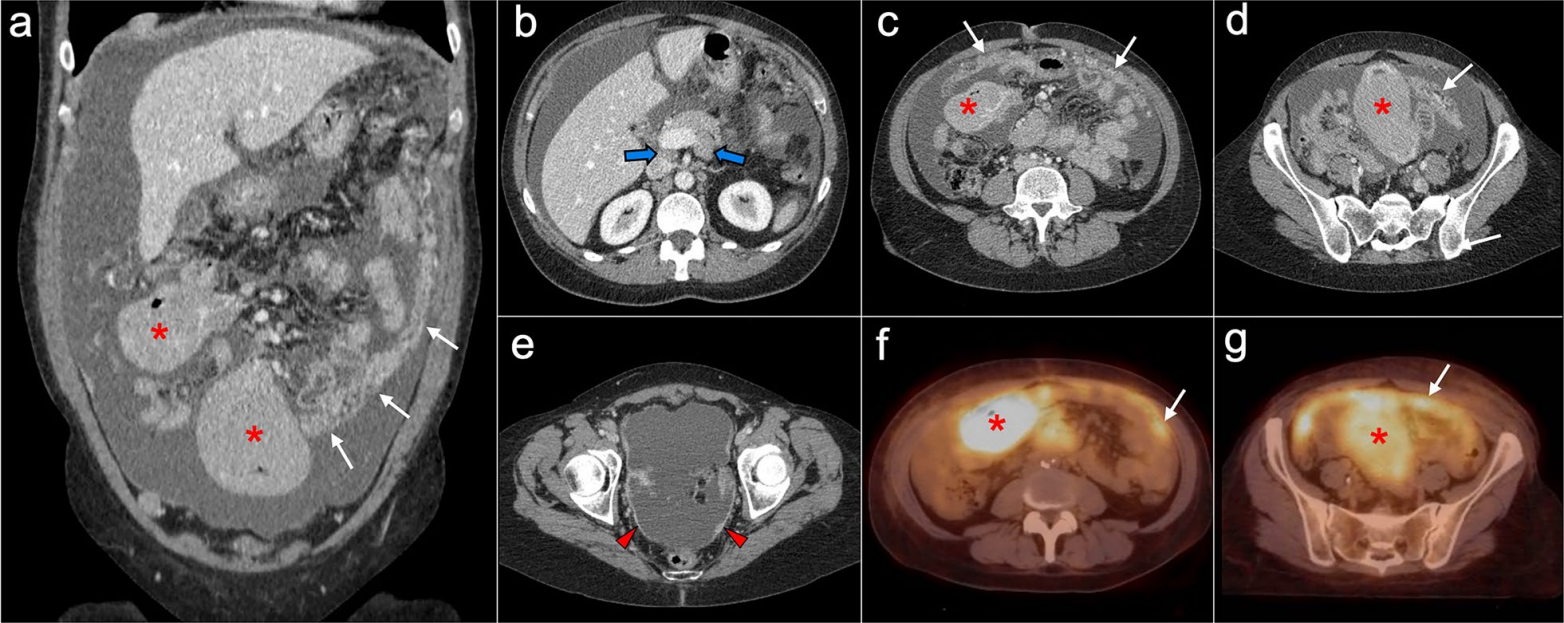
Peritoneal lymphomatosis in a 52-year-old woman, who had been complaining of abdominal distension and nausea for 3 months. Coronal (**a**) and axial (**b–e**) CT images in the portal phase show ascites, confluent nodularity of the omental fat with omental cake appearance (white arrows), thickening of the peritoneal folds (red arrowheads), retroperitoneal lymphadenopathies (blue arrows) and two bulky mesenteric masses that produce encasement of the mesenteric vessels and the adjacent small bowel loops (red asterisks). **f**, **g** Fusion PET-CT images show significant standard uptake value of the bulky masses (red asterisks) and the omental cake (white arrows). The final diagnosis of follicular non-Hodgkin's lymphoma was made by surgical biopsy of the mesenteric masses, which corresponded to adenopathic conglomerates

Imaging features on CT and MRI mimic peritoneal carcinomatosis, with a diffuse thickening of peritoneal folds, multiple omental and mesenteric nodules and masses, infiltration of small bowel mesentery and ascites. But there are some additional findings that, when present, help to reach an accurate diagnosis: mesenteric and retroperitoneal lymphadenopathies that encase mesenteric vessels, presence of mildly enhancing homogeneous bulky masses, wall thickening of long segments of small bowel loops, hepatosplenomegaly and homogeneous hepatic or splenic nodules (Figs. 25, 26) [72–76].

The use of PET/CT is recommended in patients with suspected peritoneal lymphomatosis because it improves staging, evaluation of therapy response and early detection of recurrent disease. It is also helpful in the selection of metabolically active lesions suitable for biopsy, increasing the likelihood of a diagnostic result [75].

Elevated levels of serum CA-125 as a consequence of peritoneal irritation are a common finding in peritoneal lymphomatosis and can lead to further diagnostic confusion, as this tumor marker is commonly associated with ovarian cancer [72, 74].

The final diagnosis is frequently based on pathology and a clinical history of previous lymphoma. The histology and immunophenotypic expression are the same as for lymphomas in any other location and are usually characterized by ascites containing numerous atypical lymphoid cells, with round to irregular nuclei and prominent nucleoli [3].

## Other peritoneal tumors of uncertain origin

### Desmoplastic small round cell tumor

Desmoplastic small round cell tumor (DSRCT) is a rare malignancy of unknown histogenesis that most commonly arises in the peritoneal cavity of adolescent and young adult males, with a reported mean age at diagnosis between 14 and 25 years [77–80]. It is considered a rare type of soft tissue sarcoma that belongs to the family of “small round blue cell tumors,” commonly found in the pediatric population along with neuroblastoma, malignant lymphoma, rhabdomyosarcoma, Ewing’s sarcoma, Wilms’ tumor and primitive neuroectodermal tumor [2, 77].

**Fig. 27:**
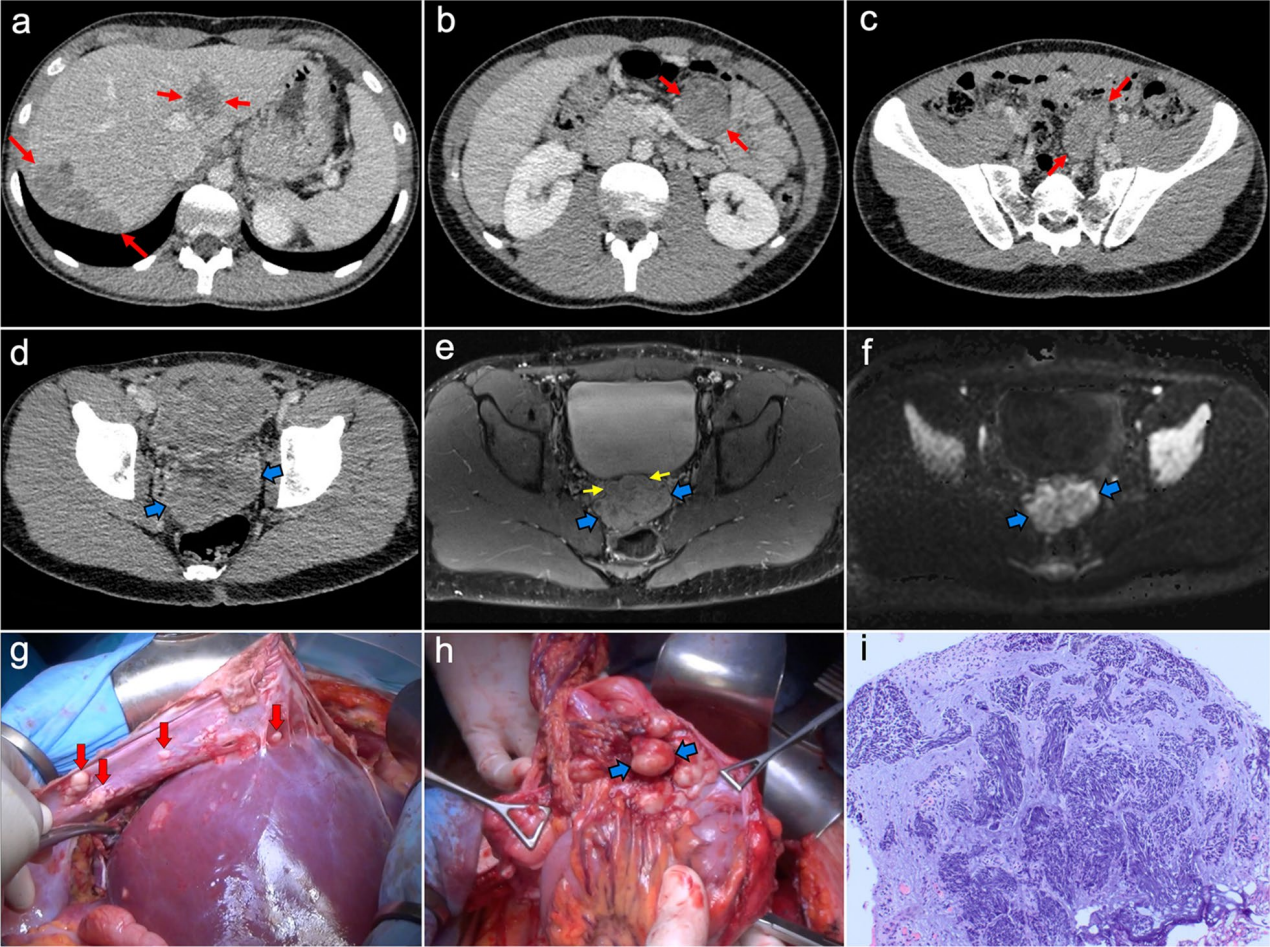
Desmoplastic small round cell tumor in a 17-year-old male, who presented with urinary frequency and a palpable pelvic mass on digital rectal examination. **a–c** Axial CT images in the portal phase show confluent implants in the right subdiaphragmatic peritoneum with infiltration of Glisson’s capsule and liver parenchyma, as well as other peritoneal implants in the hepatic round ligament and mesentery (red arrows). **d** Axial pelvic CT scan shows a bulky solid mass in the rectovesical space (blue arrows). **e** Post-contrast fat-saturated axial T1-weighted MR image obtained after neoadjuvant chemotherapy allows a better depiction of the pelvic mass (blue arrows), which shows reduction in size compared to previous CT and heterogeneous uptake with infiltration of the adjacent seminal vesicles (yellow arrows). **f** Diffusion-weighted image (b = 800) shows significant restriction of the pelvic mass. **g** Intraoperative view after stripping of the right subdiaphragmatic peritoneum, demonstrating multiple implants in this location (red arrows) that were attached to the Glisson’s capsule. **h** Intraoperative view of the pelvis shows multiple implants with a dominant mass in the rectovesical space (blue arrows). **i** H&E stain photomicrograph of the pelvic mass shows cords and nests of undifferentiated small and round malignant cells, with numerous mitotic figures and single-cell necrosis, surrounded by a dense collagenous stroma

The primary imaging findings on CT and MRI are peritoneal thickening and peritoneal nodules and masses, frequently associated with malignant ascites. The masses are characteristically heterogeneous with a sarcomatous appearance: they may contain small, punctate calcifications and usually have low-attenuation regions reflecting intratumoral necrosis or hemorrhage. Although they show a random distribution, often a bulky dominant mass may be seen, with a predilection for the retrovesical and paravesical spaces (Fig. 27). Hematogenous metastasis to the liver, lung and bone and lymphatic metastases are also common, and may occur at initial presentation [77, 78].

The histopathological features of DSRCT consist of cords and nests of undifferentiated, uniform, small and round malignant cells, surrounded by a dense collagenous stroma. The presence of numerous mitotic figures and single-cell necrosis is characteristic, as well as the identification of a reciprocal translocation t(11;22)(p13;q12) associated with the EWS-WT1 gene fusion transcript [77–80].

The treatment that has shown better results is multimodal therapy, combining CRS with systemic chemotherapy and radiation therapy. The use of intraperitoneal chemotherapy (either HIPEC or EPIC) after resection is controversial, as there is no scientific evidence of significant improvement in overall survival. Despite multimodal therapy, the prognosis is still poor, with a reported 3-year survival rate ranging from 32 to 44% and a 5-year survival rate ranging from 15 to 18% [80–82].

### Leiomyomatosis peritonealis disseminata

Leiomyomatosis peritonealis disseminata (LPD) is a very rare benign tumor, characterized by multiple nodules composed of smooth muscle cells that grow along the peritoneal cavity [83–85]. It is usually discovered incidentally in young females during surgery or imaging of uterine leiomyomas, although a few cases have also been reported in perimenopausal and postmenopausal women [86]. It is considered a type of extra-uterine leiomyomatosis, which also includes other variants such as benign metastasizing leiomyomatosis (typically of the lungs) and intravenous leiomyomatosis [87].

The etiology and pathophysiology of LPD remain unclear. The classical hormonal theory supports a spontaneous origin related to high estrogen states caused by pregnancy, oral contraceptive use, hormonal replacement therapy or estrogen-producing tumors [2, 5, 83]. Another hypothesis suggests an iatrogenic origin, since several cases have been described after surgical morcellation of uterine leiomyomas [84, 85].

**Fig. 28:**
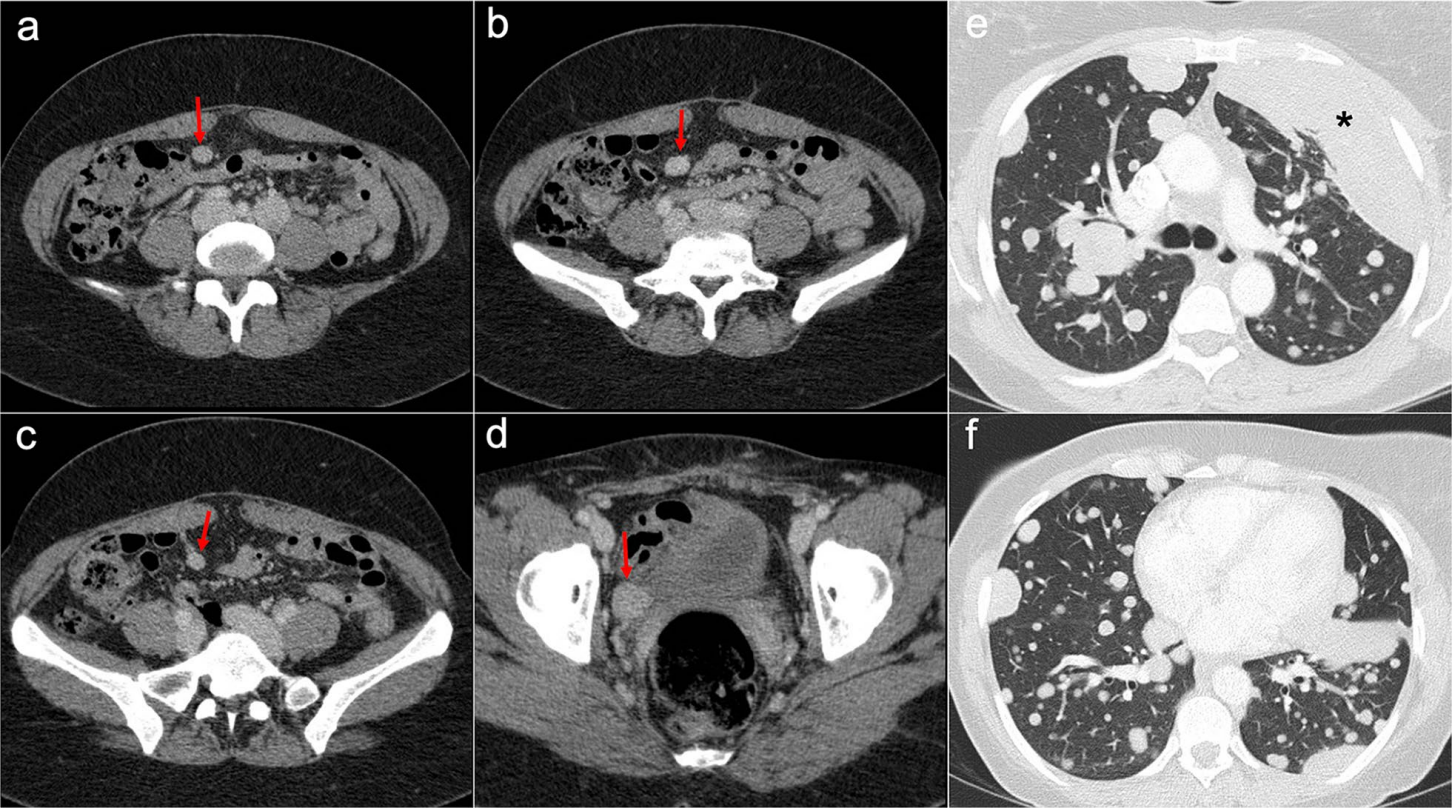
Leiomyomatosis peritonealis disseminata with associated benign metastasizing leiomyomatosis of the lung and pleura, in a 51-year-old woman with history of uterine leiomyomas and previous hysterectomy. CT was performed due to the incidental finding of multiple pulmonary nodules on chest X-ray. **a–d** Axial CT images of the abdomen in the portal phase show solid peritoneal nodules in the mesenteric root and right hemipelvis with homogenous contrast uptake (red arrows). **e**, **f** Axial CT images of the chest in the arterial phase show multiple and bilateral pulmonary and pleural solid nodules with well-defined margins, mimicking malignancy. The final diagnosis was made by histological analysis after biopsy of a pleural mass located in the lingula (**e**, black asterisk)

On CT, it presents as multiple solid nodules of well-defined margins and variable size disseminated through the peritoneum, located predominantly in the lower abdomen and with no ascites, mimicking peritoneal sarcomatosis (Fig. 28) [2, 5, 83, 84, 88]. Useful features for differential diagnosis are a relatively heterogeneous contrast uptake in the arterial phase that becomes homogenous in the portal phase, with no evidence of lymphadenopathies or visceral metastases. The behavior of the nodules on MRI resembles uterine leiomyomas and is particularly helpful: they appear isointense to muscle on T1-weighted images and show low signal on T2-weighted images [2, 5].

Currently, surgery with complete removal of the peritoneal nodules is considered the treatment of choice in LPD [82, 83], and hormone suppressive therapy is usually reserved as an alternative treatment in selected cases [85]. However, treatment should be individualized according to the patient’s hormonal and reproductive status, age and symptoms. The natural course of the disease is benign, although a few patients show a tendency to recurrence [89] and very rare cases of sarcomatous degeneration have also been reported [90].

## Miscellaneous entities that mimic peritoneal malignancy

### Granulomatous peritonitis

Granulomatous peritonitis is an unusual form of peritoneal inflammation and infection with multiple causes like infectious agents (mycobacterium tuberculosis, histoplasma, pneumocystis), sarcoidosis, foreign material (talc, barium), bowel contents, ruptured ovarian cysts or bile [3]. One of the most frequently documented etiologies is tuberculous peritonitis.

#### Tuberculous peritonitis

**Fig. 29:**
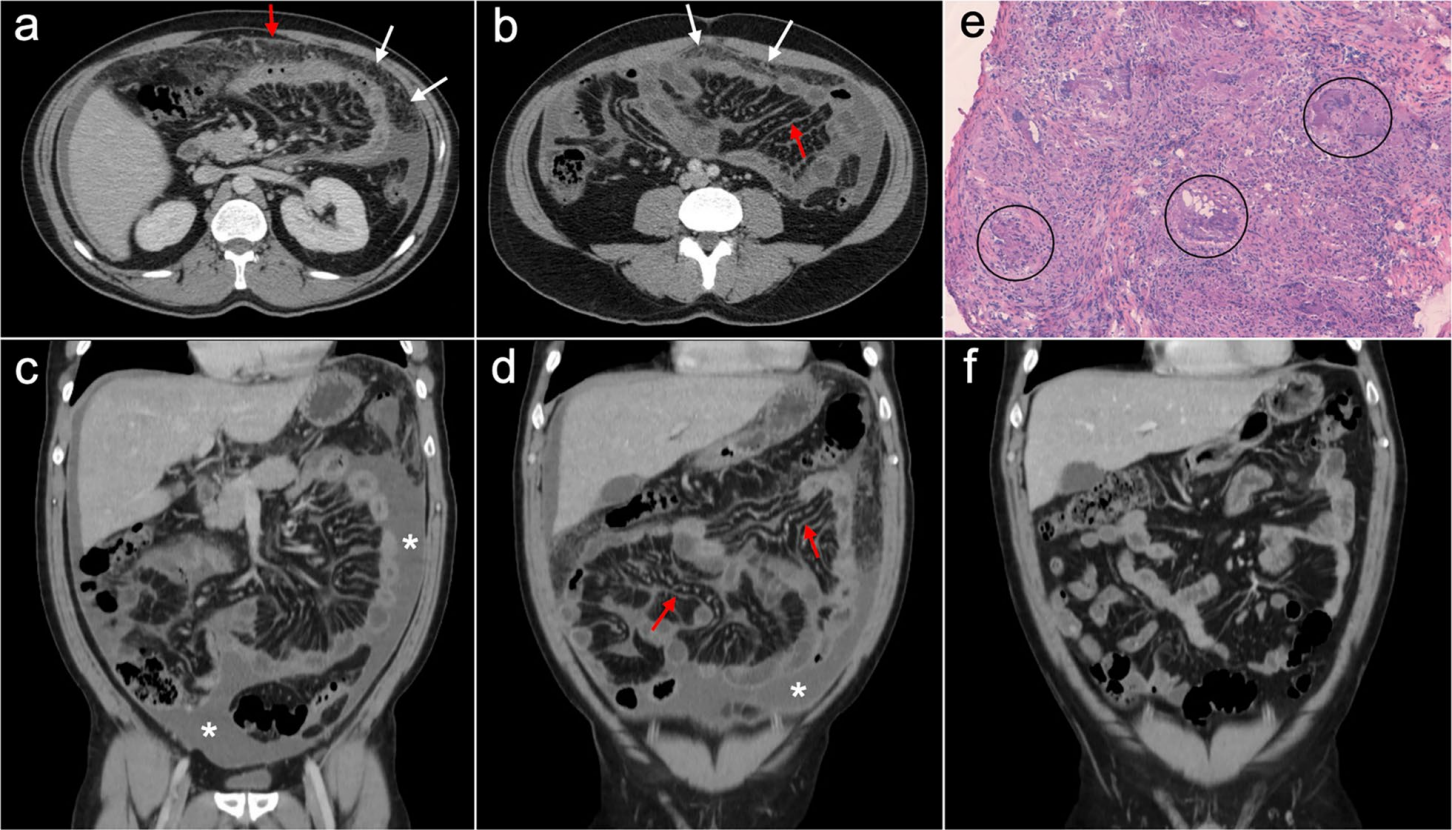
Tuberculous peritonitis in a 42-year-old male from Ecuador, presenting with constitutional syndrome, evening fever and abdominal pain. Axial (**a**, **b**) and coronal **c**, **d** CT images in the portal phase show diffuse thickening of the peritoneal folds (red arrows), stranding of the omental fat (white arrows) and ascites (white asterisks), mimicking peritoneal carcinomatosis or primary tumor of the peritoneum. Laparoscopic peritoneal biopsy was made and H&E stain photomicrograph (**e**) showed multiple granulomas with multinucleated giant cells (circles), suggesting peritoneal tuberculosis. Ascitic fluid PCR for mycobacterium tuberculosis was positive, confirming the clinical suspicion. **f** Follow-up CT scan made 6 months after tuberculostatic treatment shows a complete response, with resolution of the thickening of the peritoneal folds, the stranding of the omental fat and the ascites

It is the most common presentation of abdominal tuberculosis and involves the peritoneal cavity, the mesenterium and the omentum. On CT, it usually presents with variable imaging features that mimic peritoneal carcinomatosis, like thickening of the peritoneal folds, mesenteric and omental nodules and a variable amount of ascites (Fig. 29). The presence of a smooth and regular peritoneal thickening with pronounced enhancement is more typical of peritoneal tuberculosis, whereas nodular implants and irregular peritoneal thickening suggest peritoneal carcinomatosis. There are other additional features that may help to make a proper differential diagnosis, such as low-attenuation lymphadenopathies, lymph node calcification, hepatic or splenic microabscesses and calcifications, splenomegaly and inflammatory thickening of the ileocecal wall [91–93].

Histological analysis is often necessary to reach a definitive diagnosis and is characterized by the presence of granulomas with caseation and central necrosis [3].

Treatment is equivalent to pulmonary tuberculosis and is based on antituberculous therapy. The response is usually observed within the first 3 months of treatment and is guided by the resolution of symptoms and the normalization of laboratory values [94].

### Foreign body granuloma

Foreign body granuloma is a benign process typically seen in patients with previous surgery that can occur anywhere in the body, usually in proximity to surgical sutures. It is of clinical importance, as it often resembles a peritoneal implant or local recurrence of a previous tumor, leading to unnecessary surgical treatment [95–97].

**Fig. 30:**
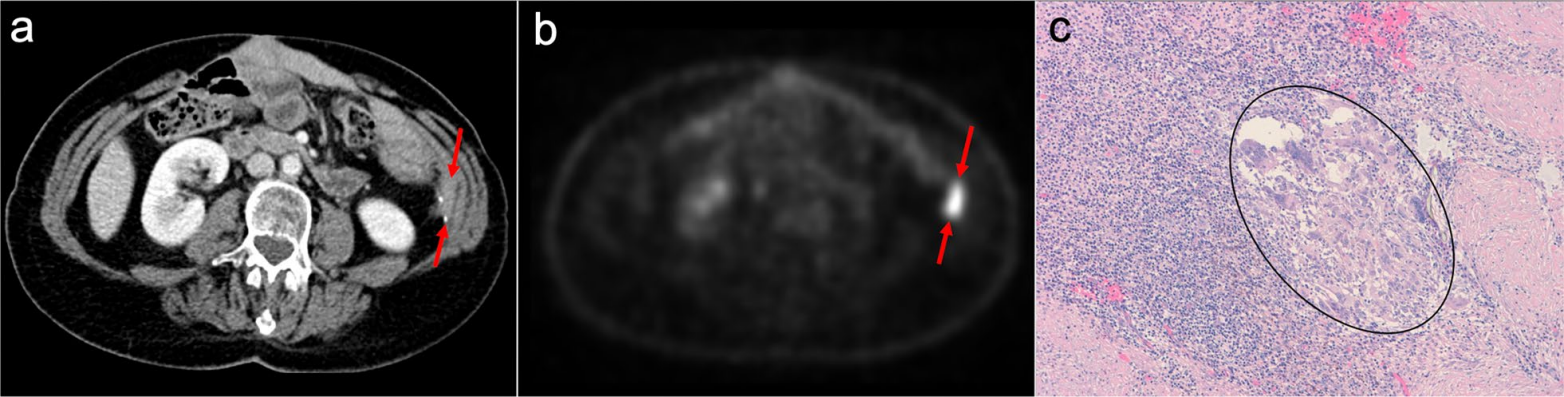
Foreign body granuloma in a 67-year-old woman with previous resection of descending colon adenocarcinoma. **a** Axial CT image in the portal phase shows a pseudonodular lesion of solid appearance in the left paracolic gutter, adjacent to surgical sutures (red arrows), that raised the suspicion of local recurrence. **b** Axial PET image showed increased metabolic activity of the lesion (red arrows). Surgical resection was made and H&E stain photomicrograph (**c**) showed a chronic inflammatory infiltrate with multinucleated giant cells of foreign body type (black oval)

It is usually visualized in postsurgical follow-up CT scans as a nodular, pseudonodular or spiculated lesion that may present progressive growth and frequently shows elevated standard uptake value on PET/CT, mimicking malignancy (Fig. 30) [95–98].

The final diagnosis is often histological, after targeted biopsy or surgical resection, and it is characterized by a chronic inflammatory infiltrate with multinucleated giant cells.

### Intra-abdominal fibromatosis

Intra-abdominal fibromatosis is a benign tumoral entity, also known as intra-abdominal desmoid tumor or mesenteric fibromatosis. It is part of the clinicopathological spectrum of deep fibromatoses, a group of benign fibroproliferative entities with locally aggressive behavior and frequent recurrences, but no metastatic potential. Small bowel mesentery is the most common site of origin. Other locations are the omentum, the ileocolic mesentery and the transverse and sigmoid mesocolon. It is associated with familial adenomatous polyposis, specifically with the Gardner syndrome variant [95].

**Fig. 31:**
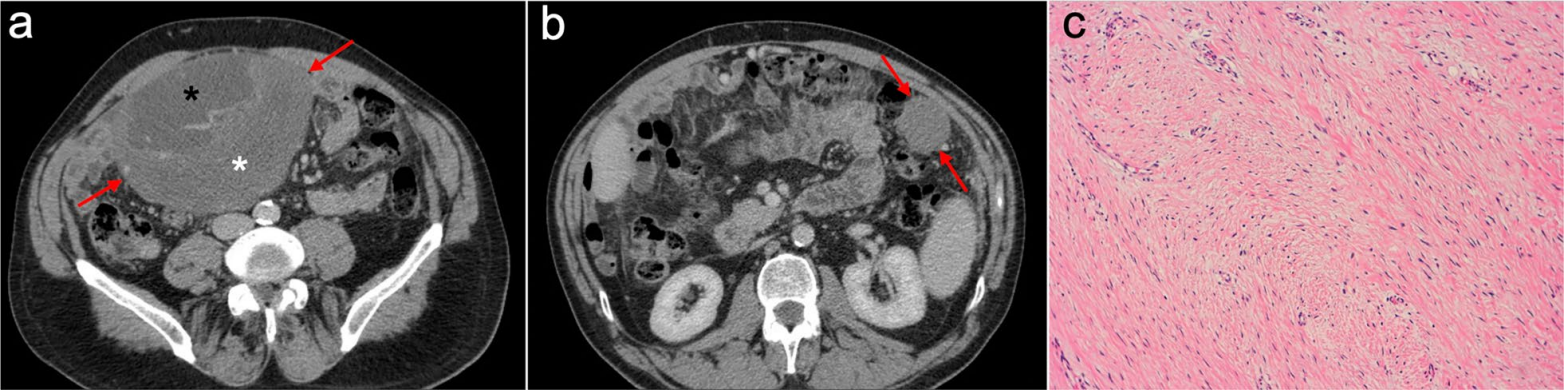
Intra-abdominal fibromatosis in a 67-year-old man. **a**, **b** Axial CT images in the portal phase show two nodular solid masses in the small bowel mesentery and the left greater omentum (red arrows). Note the mixed density of the big mesenteric mass, due to the combination of hypodense myxoid material (black asterisk) and dense collagenous material (white asterisk) within its stroma. **c** H&E stain photomicrograph shows a poorly cellular lesion without atypia or necrosis, within a background of fibrous stroma

Common imaging features are solid mesenteric or omental masses with well-defined margins and random distribution (Fig. 31). These masses may appear striated or whorled due to the alternation of areas of myxoid stroma (hypodense on CT and hyperintense on T2-weighted MR images) with collagenous areas (isodense or hyperdense on CT and hypointense on T2-weighted MR images). Predominantly myxoid lesions typically remain hypoattenuating and do not enhance with intravenous contrast. A diffuse and spiculated infiltration of the mesentery is also frequently seen, especially in Gardner syndrome [95, 99].

Histology may resemble a GIST, hence the immunohistochemical profile is key for the differential diagnosis [95]. Treatment is controversial, but many authors suggest a conservative approach due to the difficulties of complete resection, the surgical morbidity and the high recurrence rate [95].

### Inflammatory pseudotumor/inflammatory myofibroblastic tumor

Inflammatory pseudotumor (IPT) is an unusual benign chronic inflammatory lesion of unclear pathogenesis, that belongs to the group of inflammatory spindle cell lesions. For many years, it was also known as inflammatory myofibroblastic tumor (IMT), although nowadays they are regarded as separate entities: IPT is a reactive lesion that does not recur after resection and does not metastasize, whereas IMT shows a high recurrence rate after excision and a low metastatic potential [100]. IPTs and IMTs may occur in multiple sites, including the mesentery and the peritoneum [95, 101, 102]. Association with IgG4-related disease has also been described [100, 102, 103].

**Fig. 32:**
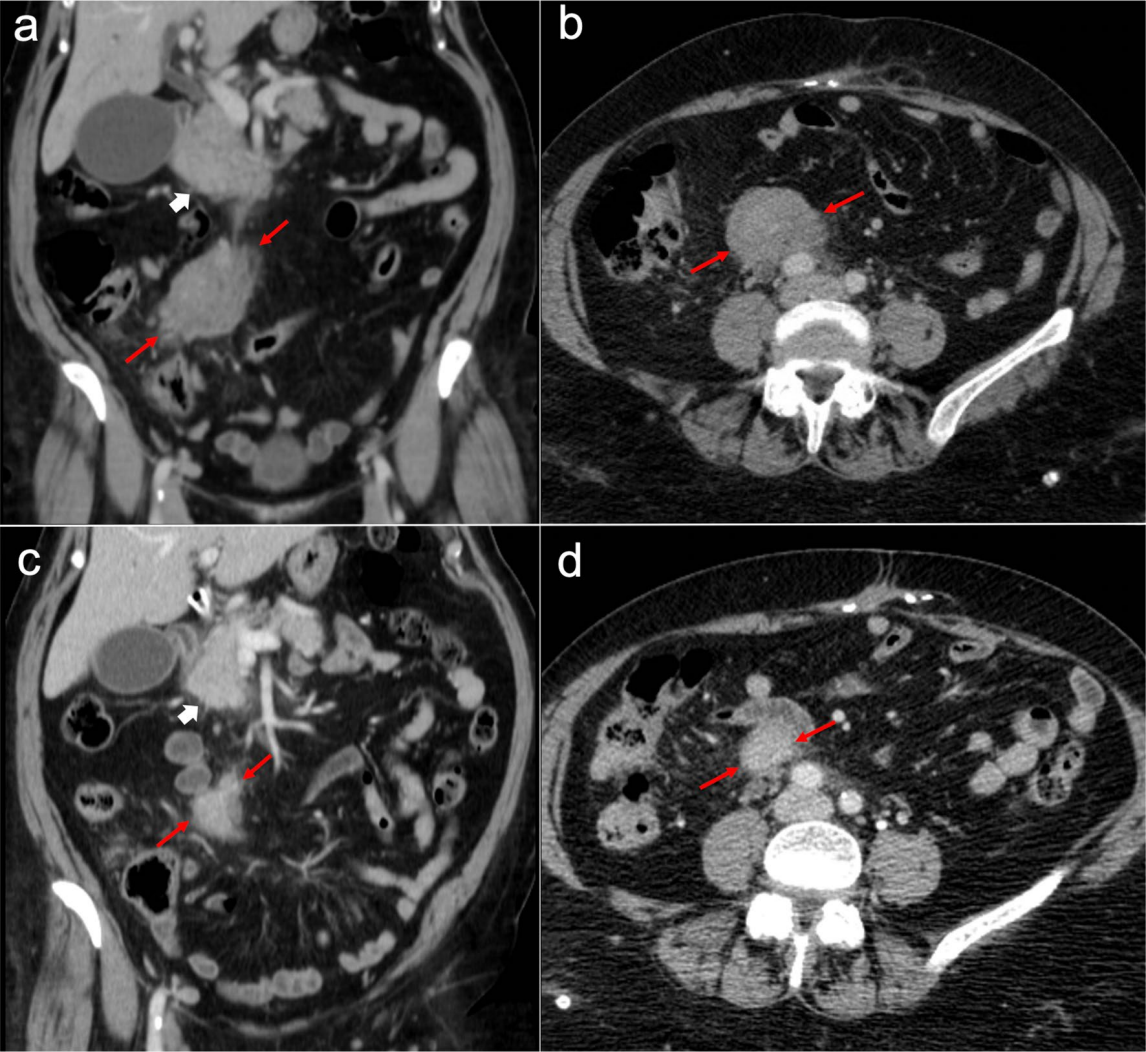
Mesenteric inflammatory pseudotumor in a 73-year-old woman with history of autoimmune pancreatitis and IgG4-related sclerosing cholangitis. Axial (**a**) and coronal (**b**) CT images in the portal phase show an increase in size of the pancreatic head secondary to autoimmune pancreatitis (white arrow) and a solid pseudonodular mass in the small bowel mesentery (red arrows). **c**, **d** Follow-up CT scan performed one month after steroids treatment shows a dramatic response, with a significant reduction in both the pancreatic head (white arrow) and the mesenteric mass (red arrows)

Both entities share similar non-specific imaging features, presenting like soft tissue masses with variable enhancement on CT, which may contain calcifications and show low signal on T2-weighted MR images (Fig. 32) [95, 100, 101, 104].

At histological analysis, IPTs and IMTs show a proliferation of fibroblasts and/or myofibroblasts accompanied by an inflammatory infiltrate of lymphocytes, plasma cells, eosinophils and histiocytes [95, 100, 102]. Differential diagnosis between them requires immunohistochemical and genetical analysis [100, 102].

Standard treatment of IPTs and IMTs is based on complete surgical resection, although conservative treatment with steroid therapy is useful in cases of IPTs associated with IgG4-related disease [100, 101, 104].

### Endometriosis

Endometriosis is a common condition that occurs in approximately 10% of young women, defined as the presence of ectopic endometrial tissue outside the uterus. It can appear in superficial locations such as the abdominal wall or the adnexal region, like the classic ovarian endometriomas. It may also present as a deep infiltrating endometriosis with fibrotic implants that produce peritoneal adhesions, causing secondary distortion and infiltration of adjacent structures such as the fallopian tubes, ovaries, pouch of Douglas, rectosigmoid and distal ileum. Deep pelvic endometriosis is more symptomatic and may cause pelvic pain, dysmenorrhea, dyspareunia, dyschezia and urinary symptoms. It is also associated with infertility [3, 105, 106].

**Fig. 33:**
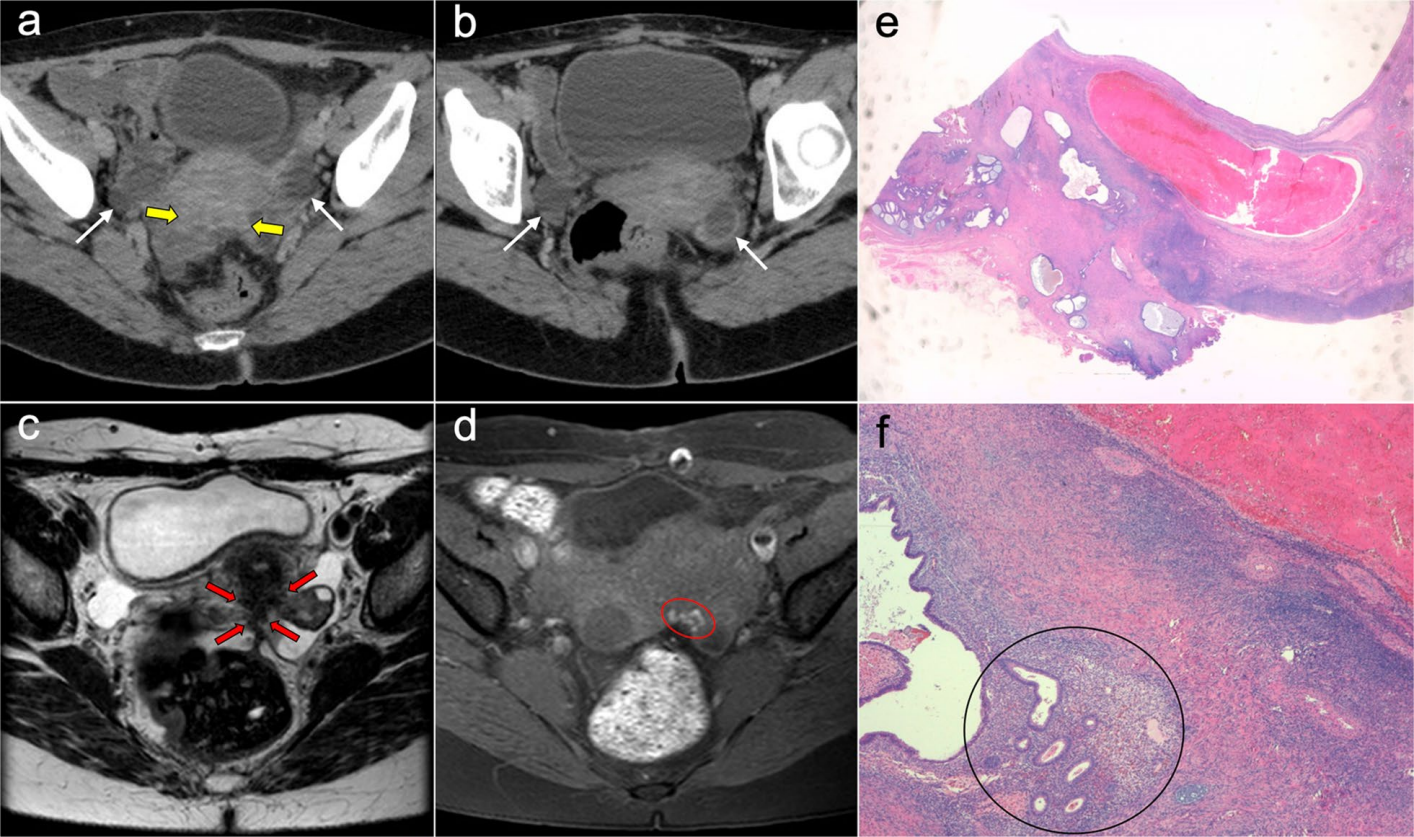
Deep pelvic endometriosis in a 37-year-old woman with previous surgery for a perforated sigmoid colon adenocarcinoma. **a**, **b** Axial CT images in the portal phase show bilateral cystic ovarian lesions (white arrows) and occupation of the pouch of Douglas by an ill-defined soft tissue lesion (yellow arrows), which raised the suspicion of pelvic recurrence. **c** Axial T2-weighted MR image shows a hypointense fibrotic endometriotic implant in the pouch of Douglas, which infiltrates and retracts the posterior uterine wall, the adnexa and the anterior rectal wall (red arrows). **d** Axial fat-suppressed T1-weighted MR image shows small hemorrhagic foci inside the fibrotic implant (red oval). An exploratory laparotomy was performed to resect the deep endometriotic implants and rule out malignancy. **e**, **f** H&E stain photomicrographs showed endometrial tissue with hemorrhagic foci and histiocytes infiltrating the mesosalpinx (black circle), confirming the diagnosis of deep pelvic endometriosis

On CT, endometriosis presents as non-specific solid, cystic or mixed pelvic masses, which in some cases may mimic peritoneal implants or local recurrence of a previous tumor. MRI is key for diagnosis, showing hyperintense hemorrhagic foci on T1-weighted images and the classical “shading” on T2-weighted images, reflecting chronic and recurrent hemorrhage. Hypointense fibrotic implants on T2-weighted images are also a common finding in deep pelvic endometriosis (Fig. 33) [3, 105, 106].

At histological analysis, endometriotic implants are defined by the presence of functional endometrial glands and stroma with hemosiderin-laden macrophages [3].

Laparoscopy is the standard of reference for the final diagnosis and treatment [105, 106].

### Splenosis

Splenosis is defined as an autotransplantation of viable splenic tissue throughout different anatomical compartments of the body after traumatic or iatrogenic rupture of the spleen. Therefore, it is an acquired condition that represents a separate entity from accessory spleens, which are congenital and result from a failure of spleen tissue fusion during embryogenesis [107, 108]. Implants of splenosis can be found anywhere in the abdominal cavity and may mimic malignant peritoneal implants, but in most cases an adequate assessment of the clinical context, looking for a previous history of splenectomy, will allow a correct diagnosis [5].

**Fig. 34:**
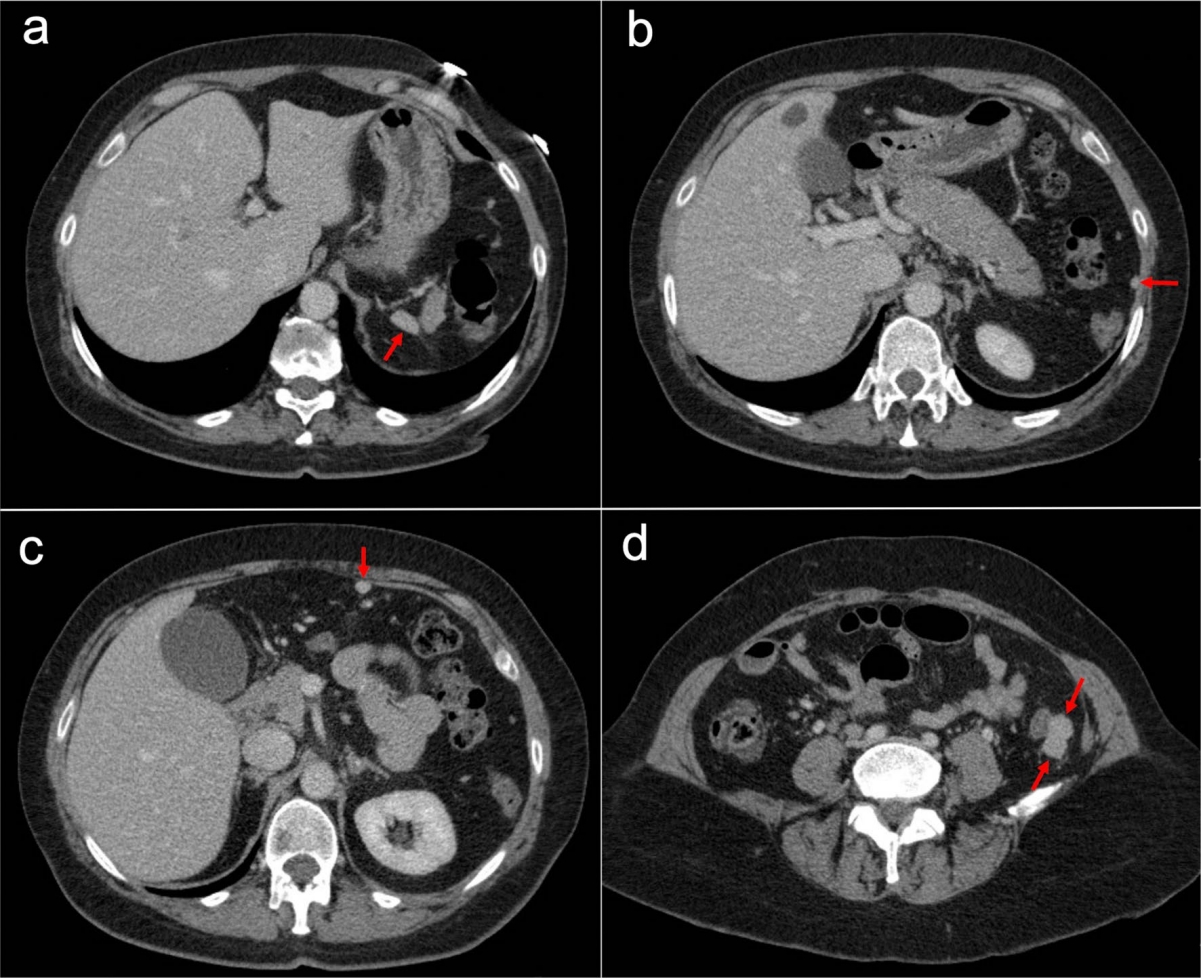
Incidental splenosis in a 50-year-old woman with history of previous splenectomy and abdominal discomfort. **a–d** Axial CT images in the portal phase show solid peritoneal nodules in the splenic fossa and left paracolic gutter, with homogeneous contrast uptake (red arrows). The distribution and characteristics of the lesions was consistent with splenosis implants and there were no other pathological findings in the rest of the abdomen. No further imaging was considered necessary

On CT, splenosis implants show an enhancement pattern identical to the normal spleen, with heterogeneous uptake in the arterial phase that becomes homogeneous during the portal phase (Fig. 34). On MRI, they also show the same signal as the normal spleen in all sequences [2, 5]. In case of doubt, Technetium-99 m-labeled heat-damaged red blood cells scintigraphy is the technique of choice to confirm the diagnosis, due to its high sensitivity and specificity to detect splenic tissue [107, 108].

Since splenosis is often asymptomatic, diagnosis is usually made incidentally and should not lead to aggressive management [5].

## Summary and conclusions

**Fig. 35:**
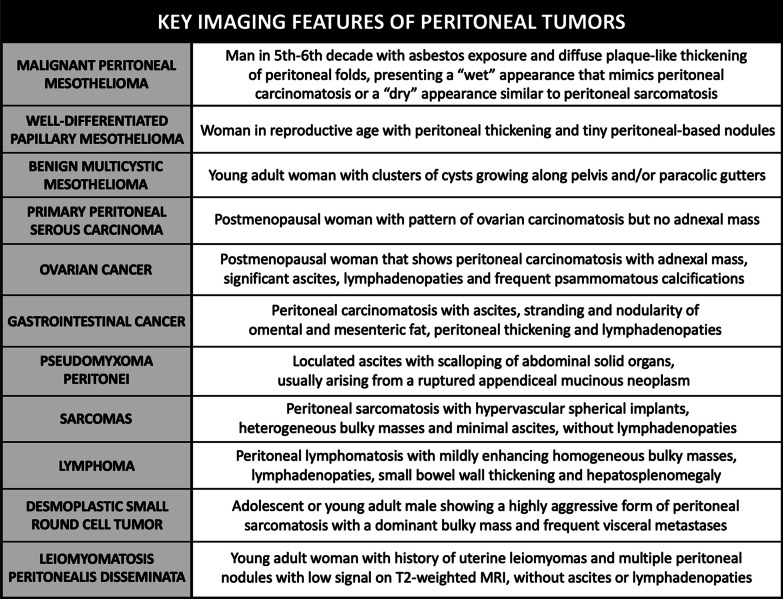
Summary of the key imaging features of peritoneal tumors

**Fig. 36:**
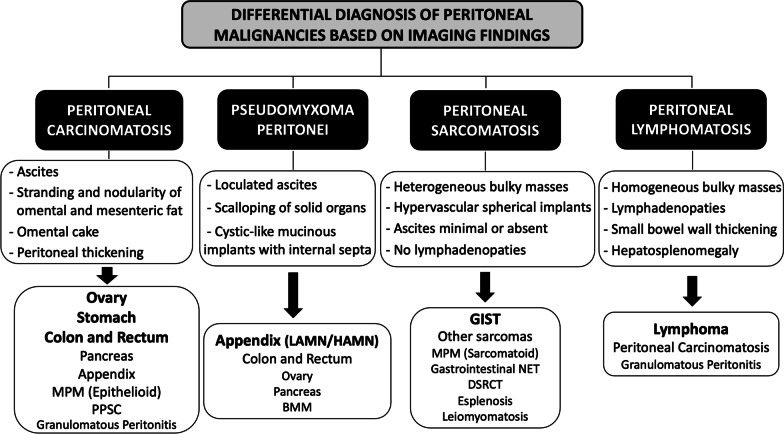
Differential diagnosis of peritoneal malignancies based on imaging findings. MPM = Malignant Peritoneal Mesothelioma, PPSC = Primary Peritoneal Serous Carcinoma, LAMN = Low-Grade Appendiceal Mucinous Neoplasm, HAMN = High-Grade Appendiceal Mucinous Neoplasm, BMM = Benign Multicystic Mesothelioma, NET = Neuroendocrine Tumor, DSRCT = Desmoplastic Small Round Cell Tu

Characterization of peritoneal tumors is a diagnostic challenge, since there is an overlap of radiological features between primary and secondary tumors of the peritoneum, and there are also benign entities that may mimic peritoneal malignancy. Therefore, histological analysis may be required to reach a final diagnosis. However, with a known primary neoplasm and in the appropriate context, imaging might be sufficient to establish a certain diagnosis. When the primary tumor is unknown, we must take into account different imaging features like the density and morphology of the implants, as well as its size, distribution and vascularization, which together with the clinical context and the age and gender of the patient will allow us to make an accurate differential diagnosis (Figs. 35, 36).

Although CT is still considered the primary technique for the initial evaluation of peritoneal tumors due to its widespread availability, peritoneal MRI is taking on an increasingly prominent role in staging and surveillance. PET/CT is also useful for ruling out nodal and extraperitoneal disease and detecting recurrences, and ultrasound is an optimal technique for image-guided biopsy.

In conclusion, imaging always plays an essential role in the assessment of peritoneal malignancies, evaluating their extension and detecting unfavorable sites of involvement that may preclude an optimal CRS, thus helping oncologists and surgeons to make an adequate therapeutic approach.

## Data Availability

All data generated or analyzed during this study are included in this published article.

## Abbreviations

AMN: Appendiceal mucinous neoplasm
BMM: Benign multicystic mesothelioma
CRS: Cytoreductive surgery
CT: Computed tomography
DSRCT: Desmoplastic small round cell tumor
FDG: Fluorodeoxyglucose
GIST: Gastrointestinal stromal tumor
H&E: Hematoxylin–eosin
HAMN: High-grade appendiceal mucinous neoplasm
HIPEC: Hyperthermic intraperitoneal chemotherapy
IMT: Inflammatory myofibroblastic tumor
IPT: Inflammatory pseudotumor
LAMN: Low-grade appendiceal mucinous neoplasm
LPD: Leiomyomatosis peritonealis disseminata
LPS: Liposarcoma
MPM: Malignant peritoneal mesothelioma
MRI: Magnetic resonance imaging
NET: Neuroendocrine tumor
PCI: Peritoneal cancer index
PET: Positron emission tomography
PMP: Pseudomyxoma peritonei
PPSC: Primary peritoneal serous carcinoma
WDPM: Well-differentiated papillary mesothelioma
